# Isolation and characterization of novel acetogenic strains of the genera *Terrisporobacter* and *Acetoanaerobium*

**DOI:** 10.3389/fmicb.2024.1426882

**Published:** 2024-07-03

**Authors:** Tim Böer, Miriam Antonia Schüler, Alina Lüschen, Lena Eysell, Jannina Dröge, Melanie Heinemann, Lisa Engelhardt, Mirko Basen, Rolf Daniel, Anja Poehlein

**Affiliations:** ^1^Genomic and Applied Microbiology and Göttingen Genomics Laboratory, Institute of Microbiology and Genetics, Georg-August-University Göttingen, Göttingen, Germany; ^2^Microbiology, Institute of Biological Sciences, University of Rostock, Rostock, Germany

**Keywords:** acetogen, *Terrisporobacter*, *Acetoanaerobium*, ethanol, prophage, Wood-Ljungdahl pathway

## Abstract

Due to their metabolic versatility in substrate utilization, acetogenic bacteria represent industrially significant production platforms for biotechnological applications such as syngas fermentation, microbial electrosynthesis or transformation of one-carbon substrates. However, acetogenic strains from the genera *Terrisporobacter* and *Acetoanaerobium* remained poorly investigated for biotechnological applications. We report the isolation and characterization of four acetogenic *Terrisporobacter* strains and one *Acetoanaerobium* strain. All *Terrisporobacter* isolates showed a characteristic growth pattern under a H_2_ + CO_2_ atmosphere. An initial heterotrophic growth phase was followed by a stationary growth phase, where continuous acetate production was indicative of H_2_-dependent acetogenesis. One of the novel *Terrisporobacter* isolates obtained from compost (strain COM^T^) additionally produced ethanol besides acetate in the stationary growth phase in H_2_-supplemented cultures. Genomic and physiological characterizations showed that strain COM^T^ represented a novel *Terrisporobacter* species and the name *Terrisporobacter vanillatitrophus* is proposed (=DSM 116160^T^ = CCOS 2104^T^). Phylogenomic analysis of the novel isolates and reference strains implied the reclassification of the *T. petrolearius*/*T. hibernicus* phylogenomic cluster to the species *T. petrolearius* and of the *A. noterae/A. sticklandii* phylogenomic cluster to the species *A. sticklandii*. Furthermore, we provide first insights into active prophages of acetogens from the genera *Terrisporobacter* and *Acetoanaerobium*.

## 1 Introduction

The genus *Terrisporobacter* was described in 2014 with *Terrisporobacter glycolicus* as the type species (Gerritsen et al., 2014). *T. glycolicus* DSM 1288^T^ (formerly, *Clostridium glycolicum*) was the first *Terrisporobacter* isolate obtained in 1962 from the mud of a stagnant pond near the Chesapeake and Ohio canal (United States). Cells were described as strict anaerobic, motile, Gram-positive and endospore-forming rods. The bacterium was of particular interest due to its ethylene glycol (Gaston and Stadtman, 1963) and cinnamic acid degrading capabilities (Chamkha et al., 2001). In 1991, the first acetogenic *Terrisporobacter* species was described, *Terrisporobacter mayombei* DSM 6539^T^ (formerly, *Clostridium mayombei*). The bacterium was isolated from the gut of the soil-feeding termite *Cubitermes speciosus* and was shown to grow acetogenically with H_2_ + CO_2_ (Kane et al., 1991). In addition to *T. mayombei*, the only other acetogenic *Terrisporobacter* isolates described comprised *T. glycolicus* DSM 13865 isolated from sewage sludge (Ohwaki and Hungate, 1977) and ATCC 29797 isolated from roots of sea grass (Küsel et al., 2001). The species *Terrisporobacter petrolearius* JCM 19845^T^, isolated in 2015 from a petroleum reservoir sample from an oilfield in Shengli (China) (Deng et al., 2015), and *Terrisporobacter hibernicus* NCTC 14625^T^, isolated in 2023 from bovine feces, were both described as non-acetogenic species (Mitchell et al., 2023). The genus *Terrisporobacter* has been found strongly associated with manure (Chen et al., 2022) and was shown to represent a key player in the degradation of manure by fermenting carbohydrates to H_2_ + CO_2_ and acetate (Budianto and Yasin, 2024). Correspondingly, biotechnological applications of bacteria from the genus *Terrisporobacter* have focused on their H_2_-producing capabilities. In contrast, biotechnological applications based on acetogenesis have hitherto been neglected. As CO is not being utilized by acetogenic *Terrisporobacter* strains an application as biocatalyst for the transformation of CO-rich industrial waste gases is unlikely. Bacteria from the genus *Terrisporobacter* are most likely to be employed as biocatalysts in the process of microbial electrosynthesis (MES) similar to the acetogenic genus *Sporomusa* (Aryal et al., 2017; Madjarov et al., 2022). The MES biotechnology allows to fix CO_2_ via acetogenesis and to transform electricity into biocommodities by extracellular electron transfer (Thulluru et al., 2023). The electricigenic potential of *Terrisporobacter* cells was already indicated by their positive correlation to bioelectricity generation in microbial fuel cells (Li et al., 2018) and their presence at the cathode of MES cells (Wang et al., 2023). Although every *Terrisporobacter* genome sequenced to date fulfilled the genetic prerequisites for acetogenesis, the conversion of H_2_ + CO_2_ to acetate was demonstrated only for a few strains. Non-acetogenic *Terrisporobacter* strains were hypothesized to have retained the Wood-Ljungdahl pathway (WLP) as an electron sink during the fermentation of amino acids via the Stickland reaction similar to *Clostridioides difficile* (Köpke et al., 2013; Gencic and Grahame, 2020; Böer et al., 2023). The genus *Acetoanaerobium* was formed in 1985 with the description of the acetogen *Acetoanaerobium noterae* ATCC 35199^T^. The bacterium was described to ferment H_2_ + CO_2_ to acetate as the sole fermentation product (Sleat et al., 1985). The first bacterium isolated from the genus *Acetoanaerobium* was the non-acetogenic bacterium *Acetoanaerobium sticklandii* DSM 519^T^ (formerly, *Clostridium sticklandii*) in 1956 from a formate enrichment culture (Stadtman and McClung, 1957). *A. sticklandii* was reclassified in 2016 to the genus *Acetoanaerobium* (Galperin et al., 2016). In 2015, a third *Acetoanaerobium* species was described, *Acetoanaerobium pronyense*. *A. pronyense* DSM 27512^T^ was isolated from a carbonate chimney of the Prony Hydrothermal Field in Prony Bay (New Caledonia) and together with *A. sticklandii* described to not transform H_2_ + CO_2_ to acetate (Bes et al., 2015). Similar to the genus *Terrisporobacter*, the biotechnological applications of bacteria from the genus *Acetonaerobium* were restricted to the degradation of protein-rich wastes to H_2_. *A. sticklandii* was shown to ferment amino acids to acetate, butyrate, acetone, butanol and ethanol as products (Sangavai and Chellapandi, 2019). Similar to *Terrisporobacter*, *Acetoanaerobium noterae* is a potential electrotroph as it was enriched at the cathode of MES cells (Wang et al., 2023). In this study, we present novel isolates of the genus *Terrisporobacter* and *Acetoanaerobium* and assess their potential for biotechnological applications by genomic and physiological characterizations.

## 2 Materials and methods

### 2.1 Enrichment and isolation of acetogenic strains

We performed enrichments with different environmental samples comprising digested sludge sampled from a wastewater treatment plant in 2019 and 2022, horse feces, river marsh sediment and compost. Environmental samples were suspended (50% w/v) in PBS-buffer (NaCl, 8 g/L; KCl, 0.2 g/L; Na_2_HPO_4_, 1.42 g/L; KH_2_PO_4_, 0.24 g/L) by shaking at 50 rpm for 1 h on a horizontal shaker (Adolf Kühner, Birsfelden, Swiss). Enrichment cultures were inoculated with 20 μL of this solution. For the enrichments with digested sludge from 2019, horse feces and river marsh sediment, the inocula were additionally pasteurized at 80°C for 10 min. H_2_-supplemented enrichment cultures were performed with the digested sludge sampled in 2019, horse feces and marsh sediment using a modified DSM 311c medium substituting sulfate salts with chloride salts, exchanging casitone with peptone and omitting fructose. The detailed recipe of the medium is provided in Supplementary Table S1. The medium (100 mL) was dispensed in sterile anaerobic 500 mL Afnor flasks (Ochs, Bovenden, Germany). The atmosphere of the flasks was changed from N_2_/CO_2_ (80%/20%) to H_2_/CO_2_ (66%/33%) by gassing for 2 min and adding an overpressure of 2 bar. H_2_-supplemented enrichment cultures were incubated at 35°C for 7 days. Subsequently, isolates were obtained by plating 50 μL of the enrichment cultures to agar plates (1.5% w/v agar) of the modified DSM 311c medium supplemented with 60 mM glucose in an anaerobic chamber. Colonies were randomly picked after 2 days of incubation at 35°C and transferred to fresh agar plates with a dilution streak. Colonies were picked from the dilution streaks and an initial taxonomic classification of the isolates was performed by colony PCR. Therefore, single colonies were suspended in 100 μL PBS-buffer and the 16S rRNA gene sequence was amplified with a DreamTaq polymerase (Thermo Fisher Scientific, Waltham, MA, United States) using 0.5 μL of the dissolved colonies as the template and the primers 08F (5′-AGAGTTTGATCCTGGC-3′) and 1504R (5′-TACCTTGTTACGACTT-3′) following the standard instructions of the manufacturer. The PCR product was purified using the Master Pure Complete DNA & RNA Purification Kit (Epicentre, Madison, United States) and Sanger sequenced by Seqlab (Microsynth, Göttingen, Germany). Isolates identified to be affiliated to bacterial genera described to contain acetogenic strains were chosen for further investigations. Another enrichment was performed with digested sludge sampled in 2022 using the modified DSM 311c medium supplemented with additional 30 mM dimethylglycine (DMG) and omitting peptone. The enrichment with DMG was incubated at 35°C for 4 days and the isolation was performed as described for the H_2_-supplemented enrichments. The compost enrichment was conducted with Brain Heart Infusion (BHI) broth (Oxoid, Hampshire, United Kingdom) supplemented with the CDMN selective supplement (Code: SR0173) (Oxoid, Hampshire, United Kingdom) containing yeast extract (5 g/L), L-Cys-HCl (0.05 g/L), moxalactam (32 mg/L) and norfloxacin (12 mg/L) (CDMN-medium) (Aspinall and Hutchinson, 1992). The CDMN enrichment cultures were incubated at 37°C and the subsequent isolation was performed with CDMN-medium and 1.5% w/v agar performing the same dilution streak method as described for the H_2_-supplemented enrichments.

### 2.2 Genome sequencing, assembly and analysis

DNA isolation and Illumina sequencing were performed as described by Böer et al. (2023). Nanopore sequencing was performed by library preparation with 1.5 μg high-molecular weight DNA using the ligation sequencing kit 1D (SQK-LSK109) and the native barcode expansion kit (EXP-NBD104) as recommended by the manufacturer (Oxford Nanopore, Oxford, United Kingdom). Nanopore sequencing was conducted for 72 h employing the MinION device Mk1B, the SpotON flow cell R9.4.1 and the MinKNOW software (v21.6.0) following the instructions of the manufacturer (Oxford Nanopore). Demultiplexing and base calling of Nanopore sequencing data were performed with the Guppy software in HAC mode (v5.0.11). Long-read *de novo* genome assemblies were performed following the suggested Trycycler workflow for bacterial genome assemblies by Wick et al. (2021). Briefly, quality control and adapter trimming of paired-end Illumina sequences was performed with Fastp (v0.23.4) (Chen et al., 2018) and Trimmomatic (v0.39; LEADING: 3, TRAILING: 3, SLIDINGWINDOW:4:15, MINLEN:50) (Bolger et al., 2014). Porechop (v0.2.4) was used for adapter trimming of Nanopore reads, which were subsequently filtered with Filtlong (v0.2.1) to a minimal read length of 1 kb discarding the worst 5% of sequences in terms of quality. Nanopore reads were subsampled 24 times with Trycycler (v0.5.4) (Wick et al., 2021) and eight subsets each were used as input for the assemblers Flye (v2.9.2) (Kolmogorov et al., 2019), Canu (v2.2) (Koren et al., 2017) and Raven (v1.8.3) (Vaser and Šikić, 2021). Assemblies were combined to a single consensus sequence with Trycycler and the consensus sequence was polished with the full Nanopore data using Medaka (v1.10.0) and finally polished with the processed short-read sequences using Polypolish (v0.5.0) (Wick and Holt, 2022). Genomes were reorientated to start with the *dnaA* gene using the fixstart function of Circlator (v1.5.5) (Hunt et al., 2015). Genome annotations were performed with Prokka (v1.14.6) (Seemann, 2014) and quality assessment of the final genome assemblies was conducted with BUSCO (v5.5.0) (Manni et al., 2021) using the reference datasets clostridia_odb for *Terrisporobacter* isolates and clostridiales_odb for the *Acetoanaerobium* isolate. Phylogenomic analysis of isolates was performed with genomes of the genus *Terrisporobacter* and *Acetoanaerobium* using pyani (v0.2.12) (Pritchard et al., 2016) and the MUMmer alignment option (Marçais et al., 2018) determining average nucleotide identities (ANIm). Additionally, digital DNA–DNA hybridization values (dDDH) of the formula d_0_, d_4_ and d_6_ were obtained by using the Genome to Genome Distance Calculator (GGDC) (v3.0) (Meier-Kolthoff et al., 2022). Assembly accession numbers of analyzed reference genomes are listed in Supplementary Table S2. Bacterial phylogenomic trees were obtained by using the Type (Strain) Genome Server (TYGS) (Meier-Kolthoff and Göker, 2019). Unique orthologous groups (OGs) were identified by orthology analysis using Proteinortho (v6.3.0) (Lechner et al., 2011) with an identity threshold of ≥50% and an e-value of ≤1e^−10^. Unique OGs and ANIm/dDDH heatmaps were visualized in R with the ggplot2 package (v3.4.1) (Wickham, 2016). Sequencing results from the prophage activity screening were mapped on to the respective reference genomes with bowtie2 (v2.5.0) (Langmead and Salzberg, 2012) and visualized with the BRIG software (v0.95) (Alikhan et al., 2011). Mapping files from bowtie2 were converted to coverage graphs with the respective BRIG module using a window size of 1 and setting the maximum coverage graph value to 1,000 (for isolates ELB, COM and DSL2) or to 4,900 (isolate DSL). Predictions for genomic islands and prophages were added from PHASTEST (Wishart et al., 2023) and IslandViewer4 (Bertelli et al., 2017). Regions showing activity in the screening were extracted as nucleotide sequences and annotated with Pharokka (Bouras et al., 2023). Phage genomes were rotated to begin with the large terminase subunit using the corresponding Pharokka function. NCBI Virus and PhageScope were employed as phage reference databases, the latter database was also used as a tool for the taxonomy, host and lifestyle predictions of phage genomes (Wang et al., 2024). Viral phylogenomic trees were obtained by using the Virus Classification and Tree Building Online Resource (VICTOR) (Meier-Kolthoff and Göker, 2017).

### 2.3 Cultivation and physiological characterization

Obtained isolates were routinely cultivated in the modified DSM 311c medium with glucose (30 mM) and inoculated to a start OD_600_ of 0.1. Growth temperature optima of the isolates were investigated first by incubation at temperatures ranging from 10 to 55°C in 5°C intervals. NaCl and pH optima were investigated subsequently at the respective temperature optimum of the strains. NaCl optima were determined by cultivation with added NaCl concentrations ranging from 0 to 7% in 1% intervals and additionally at 0.1%. pH optima were obtained by setting the pH to 5.5 to 9 in 0.5 intervals using HCl and NaOH. Gram-staining was carried out after 12 h of incubation during the exponential growth phase following the Claus protocol (Claus, 1992). Substrate utilization was evaluated by cultivation in Hungate tubes adding substrates from sterile anaerobic stock solutions in a final concentration of 30 mM. Vanillate (5 mM), syringate (5 mM) or starch (0.5 g/L) were added to the media before autoclaving. Growth was measured from triplicate cultures in Hungate tubes after 22 h of incubation at the temperature optimum of the respective strain. Basal growth was assessed by inoculating medium without substrate as control and substrate utilization was considered positive when substrate addition yielded at least an increase in OD_600_ of 0.05. Substrates not being used in the initial 22 h of incubation were continuously assessed for four more days before substrate utilization was considered negative. Utilization of H_2_ + CO_2_ of the novel isolates was assessed by cultivation of the isolates as described for the H_2_-supplemented enrichment cultures. Cultures with 2 bar overpressure under a N_2_ + CO_2_ atmosphere were used as a control. Growth was measured after 17, 24, 41, 48, 65, 72, 89, and 96 h of incubation under the optimal physiological conditions determined for the respective strain. Fermentation products were analyzed by gas chromatography as described by Baum et al. (2024). Cellular fatty acid analysis was performed with the identification service provided by the German Collection of Microorganisms and Cell Cultures (DSMZ, Braunschweig. Germany). The prophage activity screening for spontaneously released prophages was performed with all novel isolates grown to the late exponential phase. Bacterial cells were removed by centrifugation at 2,600 g for 5 min and subsequent sterile filtration of the supernatant with a 0.45 μm filter (Sarstedt, Nümbrecht, Germany). Remaining cells and host DNA were removed by the addition of 20 μL of lysozyme (100 mg/mL) (Serva, Heidelberg, Germany), 20 μL of DNase I (1 U/μL) (Epicentre, Madison, United States) and 20 μL of RNase A (5 μg/μL) (Epicentre, Madison, United States) and incubation at 37°C overnight. The following day the solution was centrifuged at 36,000 g for 2 h at room temperature in a DU PONT OTD 50B ultracentrifuge using the Ti-60 rotor (Thermo Fisher Scientific, Waltham, United States). The supernatant was discarded and the resulting phage pellet was suspended in 100 μL of TKM-buffer (10 mM Tris–HCl (pH 7.5), 10 mM KCl and 1 mM MgCl_2_) and stored at 4°C. Prior to the DNA isolation, DNase I was inactivated by incubation at 75°C for 10 min. DNA isolation from the dissolved phage particles and sequencing was performed with the protocol as described for bacterial genome sequencing. A description of the general principle of prophage activity screenings by sequencing of particle-protected DNA can be found in Hertel et al. (2015) and Schüler et al. (2024).

## 3 Results

### 3.1 Isolates

Enrichments were performed with the inocula listed in Table 1. We obtained one isolate from each of the five different inocula used in this study. Based on the initially analyzed 16S rRNA gene sequences the isolates DSL, ELB, HSE, and COM were associated to the genus *Terrisporobacter* and the isolate DSL2 was associated to the genus *Acetoanaerobium*.

**Table 1:** Isolates obtained from the different environmental samples and the corresponding enrichment and isolation strategy.

Sample	Location	Enrichment	Isolation	Isolate
Digested sludge (pasteurized)	Digestion tower from waste water treatment plant, Göttingen, Germany 2019. (51°33′17.0″, N 9°55′07.9″E)	DSM 311c + H_2_ + CO_2_	DSM 311c agar + glucose	DSL
Digested sludge	Digestion tower from waste water treatment plant, Göttingen, Germany 2022. (51°33′17.0″, N 9°55′07.9″E)	DSM 311c (−peptone) + DMG	DSM 311c agar + glucose	DSL2
Horse feces (pasteurized)	Horse (*Equus caballus*), Gelliehausen, Germany 2020. (51°28′35.7″, N 10°02′57.5″E)	DSM 311c + H_2_ + CO_2_	DSM 311c agar + glucose	HSE
River marsh sediment (pasteurized)	Pioneer zone from low marsh to Elbe river, Hollerwettern, Germany 2020. (53°50′19.8”N 9°21′16.3″E)	DSM 311c + H_2_ + CO_2_	DSM 311c agar + glucose	ELB
Compost	Composting plant, Königsbühl, Germany 2020. (51°34′23.2”N, 9°54′32.8″E)	CDMN	BHI agar	COM

### 3.2 Genomic characterization

Genome sequencing and annotation data of the isolated organisms are summarized in Table 2. The assemblies for the isolates HSE, ELB, DSL, and COM yielded chromosomes with a GC-content of 29% and sizes ranging from 4.015–4.115 Mb. Additionally, circular plasmids with sizes ranging from 5.581–48.861 kb could be found. Assembly of the DSL2 genome yielded one circular replicon with a GC-content of 34% and a size of 2.633 Mb.

**Table 2:** Genome and annotation data of the obtained isolates.

	HSE	ELB	DSL	COM	DSL2
Genome size (bp)	4,115,567	4,031,631	4,138,706	4,015,703	2,633,942
Plasmids size (bp)	pTPHSE1 48,861 pTPHSE2 21,696	pTHELB1 21,684 pTHELB2 5,657	pTHDSL1 21,645 pTHDSL2 9,281	pTVTCOM1 21,519 pTVTCOM2 19,448 pTVTCOM3 5,581	–
GC-content (%)	29	29	29	29	34
BUSCO score	C:100.0% [S:98.8%, D:1.2%] F:0.0%, M:0.0% n:247	C:100.0% [S:98.8%, D:1.2%] F:0.0%, M:0.0% n:247	C:100.0% [S:98.8%, D:1.2%] F:0.0%, M:0.0% n:247	C:100.0% [S:98.0%, D:1.6%] F:0.4%, M:0.0% n:247	C:100.0% [S:98.9%, D:1.1%] F:0.0%, M:0.0% n:247
Genes	4,103	3,935	4,072	3,962	2,530
CDS	3,969	3,805	3,942	3,833	2,453
Functional proteins	2,076	2,030	2,051	2,047	1,468
Hypothetical proteins	1,893	1,775	1,891	1,786	985
rRNA (5S; 16S; 23S)	13, 13, 13	12, 12, 12	12, 12, 12	12, 12, 12	6, 6, 6
tRNAs	94	93	93	92	58
tmRNAs	1	1	1	1	1
CRISPR repeats	1	1	0	1	1
GenBank accession	CP154619.1–CP154621.1	CP154622.1–CP154624.1	CP154616.1–CP154618.1	CP154625.1–CP154628.1	CP154629.1
BioProject genome	PRJNA1068306	PRJNA1068312	PRJNA1068309	PRJNA1068316	PRJNA1068318
BioSample genome	SAMN39584973	SAMN39585366	SAMN39584974	SAMN39585368	SAMN39585619
Sequence Read Archive Illumina genome	SRR27707071	SRR27707817	SRR27708422	SRR27776547	SRR27708508
Sequence Read Archive Nanopore genome	SRR27707818	SRR27707819	SRR27708421	SRR27776548	SRR27708509
GenBank 16S rRNA	–	–	–	PP196406	–
BioProject prophage screening	–	PRJNA1068489	PRJNA1068491	PRJNA1070888	PRJNA1068494
BioSample prophage screening	–	SAMN39585366	SAMN39584974	SAMN39585368	SAMN39585619
Sequence Read Archive Illumina prophage screening	–	SRR27722894	SRR27722895	SRR27778306	SRR27722916
GenBank phage genome	–	PP197244 (TPELB_ph1)	PP197246 (TPDSL_ph1) PP197247 (TPDSL_ph2)	PP197245 (TVCOM_ph1)	PP197248 (ANDSL2_ph1)

#### 3.2.1 Phylogenomic analysis

**Figure 1:**
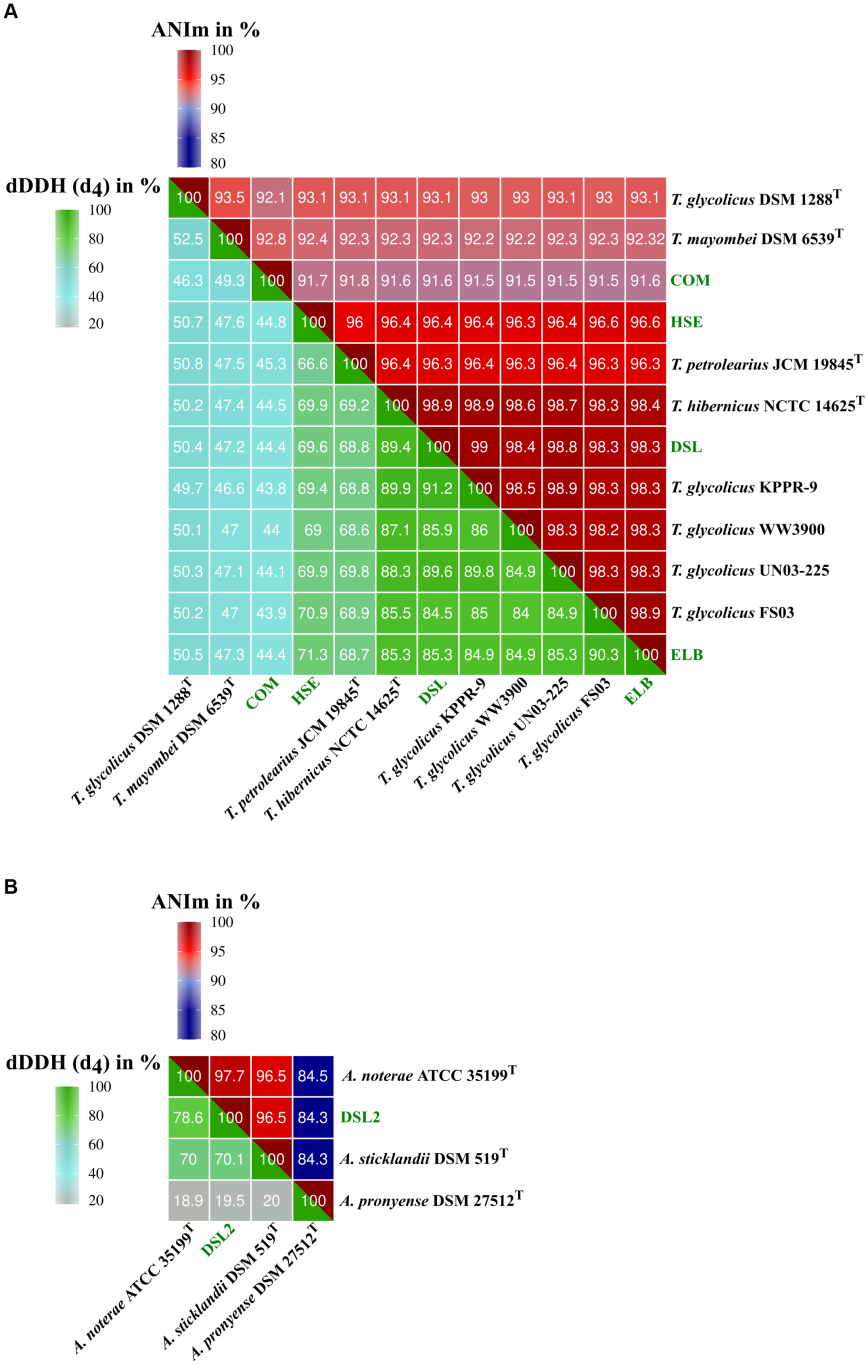
Phylogenomic analysis of novel *Terrisporobacter* and *Acetoanaerobium* isolates. The heatmaps visualize ANIm and dDDH values for the genomes of *Terrisporobacter* isolates HSE, ELB, DSL and COM **(A)** and the *Acetoanaerobium* isolate DSL2 genome **(B)**. The top part of the heatmaps visualize the ANIm percentage identity values from 80–100% and the lower part of the heatmaps visualize dDDH values (*d*_4_) from 20–100%. The legend for the color code is shown on top (ANIm) and to the left (dDDH). Novel isolates from this study are highlighted in green.

**Figure 2:**
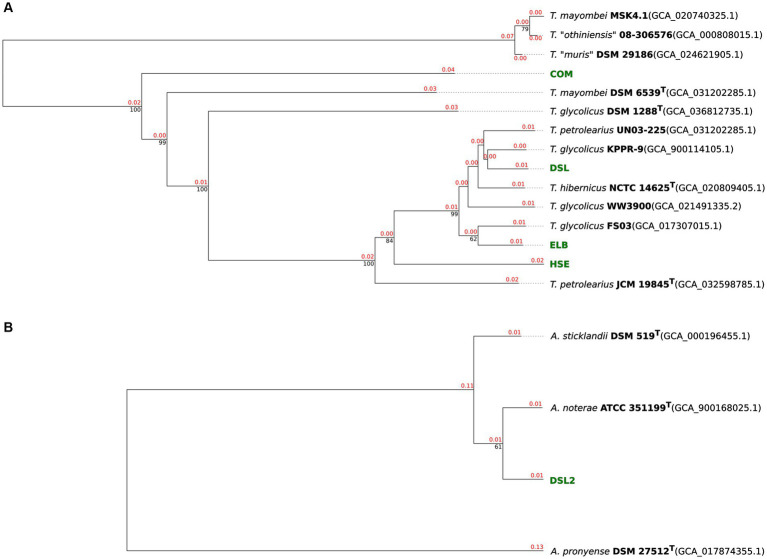
Phylograms of novel *Terrisporobacter* and *Acetoanaerobium* isolates. Whole-genome sequence-based phylogenetic tree of novel *Terrisporobacter* isolates **(A)**. Branch lengths are scaled in terms of GBDP distance formula *d*_5_. Red numbers above branches are GBDP pseudo-bootstrap support values >60% from 100 replications, with an average branch support of 73.9%. Black numbers below branches are confidence scores. The tree was rooted at the midpoint. Whole-genome sequence-based phylogenetic tree of the novel *Acetoanaerobium* isolate **(B)**. Branch lengths are scaled in terms of GBDP distance formula *d*_5_. Red numbers above branches are GBDP pseudo-bootstrap support values >60% from 100 replications, with an average branch support of 61.0%. Black numbers below branches are confidence scores. The tree was rooted at the midpoint.

The genomes of isolates HSE, ELB, DSL, and COM were compared to genome sequences of the genus *Terrisporobacter* (Figure 1A) and that of DSL2 to genomes of *Acetoanaerobium* (Figure 1B) by ANIm and dDDH (d_4_) analyses in order to obtain a phylogenomic classification. Complete GGDC results including the three dDDH formulas d_0_, d_4_, d_6_ and their respective confidence intervals are summarized in Supplementary Table S3. The ELB and DSL genomes were more identical to the type strain of *T. hibernicus* genome (ANIm: 98.4 and 98.9%, dDDH (d_4_): 85.3 and 89.4%) than to the type strain genome of *T. petrolearius* (ANIm: 96.3 and 96.3%, dDDH (d_4_): 68.7 and 68.8%). The HSE genome showed similar identities to the type strains genomes of *T. hibernicus* (ANIm: 96.4%, dDDH (d_4_): 69.9%) and *T. petrolearius* (ANIm: 96%, dDDH (d_4_): 66.6%). The genome of the COM isolate showed highest sequence identity to the type strain genome of *T. mayombei* DSM 6539^T^ (ANIm: 92.8%, dDDH (d_4_): 49.3%) and *T. glycolicus* DSM 1288^T^ (ANIm: 92.1%, dDDH (d_4_): 46.3%). The DSL2 genome was most identical to the type strain genome of *A. noterae* (ANIm: 97.7%, dDDH (d_4_): 78.6%). The genome of *A. sticklandii* DSM 519^T^ showed the highest sequence identities to that of the *A. noterae* type strain (ANIm: 96.5%, dDDH (d_4_): 70.0%) and the DSL2 isolate (ANIm: 96.5%, dDDH (d_4_): 70.1%). Whole genome-based phylograms created by TYGS identified *T. mayombei* DSM 6539^T^ as the closest relative of the COM isolate and assigned the novel *Terrisporobacter* isolates HSE, ELB and DSL in a phylogenomic group together with the strains *T. glycolicus* FS03, WW3900, KPPR-9, *T. petrolearius* UN03-225, JCM 19845^T^ and *T. hibernicus* NCTC 14625^T^ (Figure 2A). The *Acetonaerobium* isolate DSL2 was placed into a phylogenomic group with *A. noterae* ATCC 35199^T^ and *A. sticklandii* DSM 519^T^ (Figure 2B).

#### 3.2.2 Pan/core genome analysis

**Figure 3:**
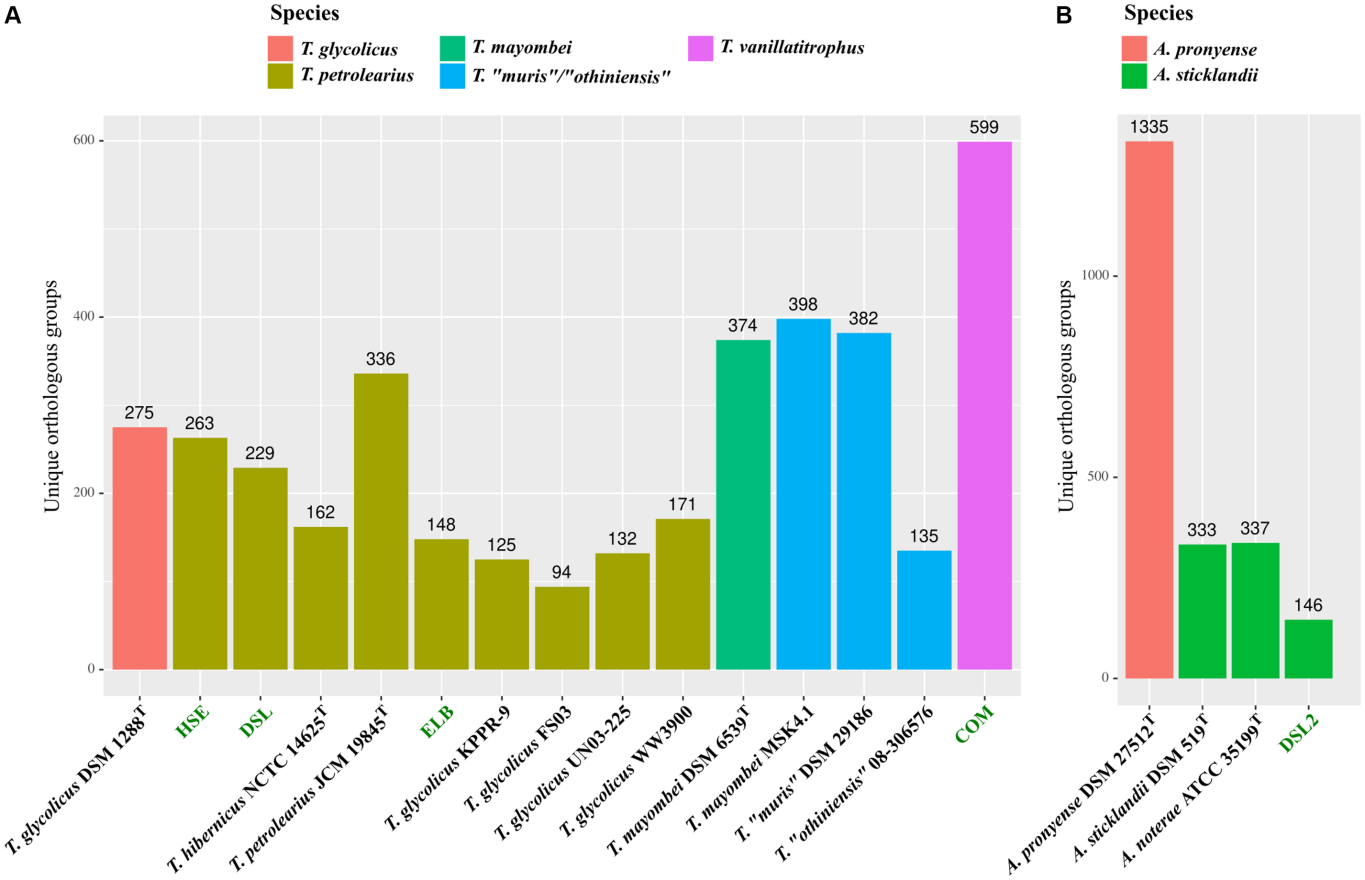
Pan/core genome analysis of novel isolates. Detected unique OGs in the novel and reference *Terrisporobacter* **(A)** and *Acetoanaerobium* **(B)** genomes colored by species. Novel isolates from this study are highlighted in green and the species color code is shown on the top.

After species assignment of all novel isolates, we performed a pan/core genome analysis to identify potential novel physiological traits. Encoded proteins of *Terrisporobacter* reference genomes were clustered into orthologous groups (OGs) and compared to the novel *Terrisporobacter* isolates HSE, ELB, DSL, and COM (Figure 3A). Similarly, the OGs of encoded proteins by *Acetoanaerobium* reference genomes were compared to those of the DSL2 genome (Figure 3B). OGs detected in only one genome were assigned as unique OGs and colored corresponding to the species assignments of our phylogenomic classification. The genus *Terrisporobacter* formed a pan genome of 9,867 OGs with a core genome of 800 OGs. All *Terrisporobacter* genomes encoded a WLP-gene cluster, a potential HDCR-complex, an electron-bifurcating hydrogenase (HydABC), an electron-bifurcating *Sporomusa*-type Nfn transhydrogenase (StnABC) and a Rnf-complex (RnfABCDEG). The highest number of unique OGs were detected for isolate COM (599), followed by *T. mayombei* MSK4.1 (398), *T. “muris”* DSM 29186 (382), *T. petrolearius* JCM 19845^T^ (336), *T. glycolicus* DSM 1288^T^ (275), isolate HSE (263) and isolate DSL (229). The lowest number of unique OGs were identified for *T. glycolicus* WW3900 (171), *T. hibernicus* NCTC14625^T^ (162), isolate ELB (148), *T. “othiniensis”* 08-306,576 (135), *T. petrolearius* UN03 (132), *T. glycolicus* KPPR-9 (125) and *T. glycolicus* FS03 (94). The genome of isolate COM contained unique OGs for ArgBCDJ (TVTCOM_02650-02680), as well as ArgG (TVTCOM_21250), ArgF (TVTCOM_20250) and ArgH (TVTCOM_31940). Furthermore, four unique OGs were identified encoding the subunits PorABCD (TVTCOM_25810-25840) of a pyruvate ferredoxin oxidoreductase (PFOR). Other unique OGs encoded by the COM genome comprised subunits of a dissimilatory sulfite reductase (TVTCOM_04940-04960), proteins for the utilization of mannitol (TVTCOM_07370-07410) and 1,2-propanediol (TVTCOM_08460-08620), the subunits GrdH and GrdI of a betaine reductase (TVTCOM_13260-13270), proteins for the import of aliphatic sulfonates (TVTCOM_13580-13600 and TVTCOM_33140-33160), and proteins for the import and export of siderophores for the acquisition of iron (TVTCOM_18490-18530). The plasmid pTVTCOM2 encoded unique *uviA* and *uviB* genes (TVTCOM_39450-39460). The pan genome of the genus *Acetoanaerobium* comprised 4,591 OGs including the core genome of 1,387 OGs. All *Acetoanaerobium* genomes encoded an electron-bifurcating hydrogenase with three subunits (HydABC), a Rnf-complex and a PFOR gene. With the exception of *A. pronyense* DSM 27512^T^, every genome encoded a WLP-gene cluster and an electron-bifurcating *Sporomusa*-type Nfn transhydrogenase (StnABC). The highest number of unique OGs was identified for *A. pronyense* DSM 27512^T^ (1,335) followed by *A. noterae* ATCC 35199^T^ (337), *A. sticklandii* DSM 519^T^ (333) and isolate DSL2 (146).

### 3.3 Physiological characterization

All novel isolates grew only under strict anoxic conditions and no growth occurred when yeast extract was omitted. Growth of the isolates HSE, ELB, DSL and COM was also considerably improved by the addition of peptone. The rod-shaped cells stained Gram-positive and endospore formation was observed by light microscopy. In contrast, the rod-shaped cells of the DSL2 isolate stained Gram-negative and endospore formation was not detected by light microscopy. Cellular fatty acid analysis of isolate COM is summarized in Supplementary Table S4. The predominant cellular fatty acids identified were C16:0 (19%), C16:0 DMA (12.4%), C16:1 ∆9 (7.3%) and C16:1 ∆7 (6%).

#### 3.3.1 Growth optima and substrate utilization

Isolate-specific growth optima are shown in Table 3. All isolates grew at temperatures between 20–50°C. HSE and ELB showed the lowest temperature optimum at 25°C, while the DSL isolate grew best at 30°C. The highest temperature optima were determined for the COM and DSL2 isolate at 35°C. The DSL and DSL2 isolates tolerated added NaCl concentrations between 0 to 5% and 0 to 7%, thereby showing the broadest range of NaCl tolerance. However, all isolates grew optimally at low added NaCl concentrations ranging from 0 to 0.1%. With a pH of 8.5 and 8, the HSE and ELB isolate showed the highest pH optima in comparison to the other isolates growing optimally at pH 7 and 7.5. The substrate utilization tests yielded specific characteristics for every isolate (Table 4). The isolates HSE, ELB, DSL and COM all utilized fructose, glucose, sorbitol, maltose, raffinose and melezitose. None of these isolates utilized arabinose, ribose, galactose, mannose, rhamnose, glucuronic acid, myo-inositol, lactose, melibiose, dextran, starch, pyruvate, lactate, malate, citrate, DMG, betaine, syringate, methanol or ethanolamine. ELB was the only isolate utilizing trehalose for growth, while DSL was the only isolate growing with glycerol. Xylose fermentation was only observed for the DSL and COM isolates. Mannitol, cellobiose, formate, vanillate and 1,2-propanediol utilization were identified as unique substrates utilized by the COM isolate. Glucose was fermented to acetate and ethanol as the only fermentation products by the COM isolate. The isolate DSL2 utilized glucose, maltose, fructose, sorbitol, sucrose, trehalose, melibiose, raffinose, melezitose and dextrin for growth.

**Table 3:** Determined physiological optima of the novel *Terrisporobacter* isolates HSE, ELB, DSL, and COM and the novel *Acetoanaerobium* isolate DSL2.

	HSE	ELB	DSL	COM	DSL2
Temperature optimum	25°C	25°C	30°C	35°C	35°C
Temperature range	20–45°C	20–45°C	20–40°C	20–40°C	35–50°C
NaCl optimum	0%	0%	0.1%	0.1%	0.1%
NaCl range	0–3%	0–2%	0–5%	0–3%	0–7%
pH optimum	8.5	8	7	7.5	7

**Table 4:** Substrate utilization of the novel *Terrisporobacter* isolates HSE, ELB, DSL, and COM and the novel *Acetoanaerobium* isolate DSL2.

Substrate	HSE	ELB	DSL	COM	DSL2
L(+)-Arabinose	−	−	−	−	−
D(−)-Ribose	−	−	−	−	−
D(+)-Xylose	−	−	+	+	−
D(−)-Fructose	+	+	+	+	+
D(+)-Galactose	−	−	−	−	−
D(+)-Glucose	+	+	+	+	+
D(+)-Mannose	−	−	−	−	−
L(+)-Rhamnose	−	−	−	−	−
D(−)-Glucuronic acid	−	−	−	−	−
Glycerol	−	−	+	−	−
D(−)-Sorbitol	+	+	+	+	+
D(−)-Mannitol	−	−	−	+	−
*myo*-Inositol	−	−	−	−	−
D(+)-Sucrose	+	+	−	−	+
D(+)-Cellobiose	−	−	−	+	−
D(+)-Trehalose	−	+	−	−	+
D(+)-Lactose	−	−	−	−	−
D(+)-Maltose	+	+	+	+	+
D(+)-Melibiose	−	−	−	−	+
D(+)-Raffinose	+	+	+	+	+
D(+)-Melezitose	+	+	+	+	+
Dextran	−	−	−	−	−
Dextrin	+	+	+	+	+
Starch	−	−	−	−	−
Formate	−	−	−	+	−
Pyruvate	−	−	−	−	−
DL-Lactate	−	−	−	−	−
DL-Malate	−	−	−	−	−
Citrate	−	−	−	−	−
DMG	−	−	−	−	−
Betaine	−	−	−	−	−
Vanillate	−	−	−	+	−
Syringate	−	−	−	−	−
Methanol	−	−	−	−	−
1,2-propanediol	−	−	−	+	−
Ethanolamine	−	−	−	−	−

+, positive; −, negative.

#### 3.3.2 Acetogenesis in H_2_-supplemented cultures

**Figure 4:**
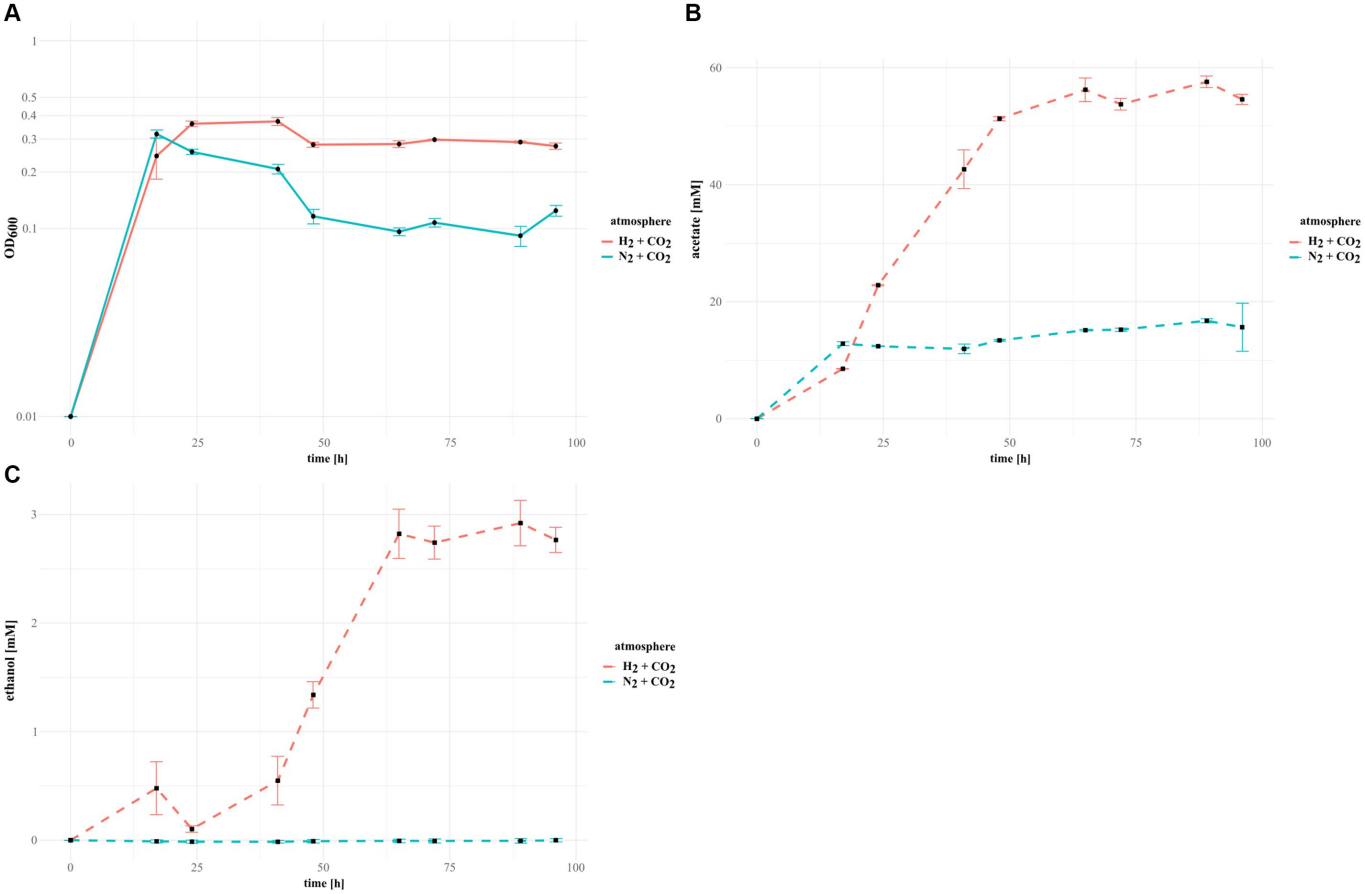
Growth in H_2_-supplemented cultures and control cultures of the novel *Terrisporobacter* isolate COM. Measured OD_600_ growth values for COM cultures grown with an atmosphere of H_2_ + CO_2_ (solid red line) and control cultures with an atmosphere of N_2_ + CO_2_ (solid blue line) **(A)**. GC-measurements detecting the amount of produced acetate **(B)** and ethanol **(C)** in H_2_ + CO_2_ (dotted red line) and N_2_ + CO_2_ (dotted blue line) cultures.

The *Terrisporobacter* isolates HSE, DSL and COM as well as the *Acetoanaerobium* isolate DSL2 grew in the initial 17 h of incubation faster in the N_2_ + CO_2_ control cultures than under an H_2_ + CO_2_ atmosphere. Only the *Terrisporobacter* isolate ELB achieved a higher OD_600_ after 17 h with H_2_ + CO_2_ than in the control. Nevertheless, all isolates achieved their maximal OD_600_ within 48 h of incubation under H_2_ + CO_2_ and the N_2_ + CO_2_ control (Figure 4; Supplementary Figure S1). The *Terrisporobacter* isolates HSE, ELB, DSL and COM achieved higher maximal growth in H_2_ + CO_2_ cultures (OD_600_: 0.401, 0.345, 0.442 and 0.37) than in control cultures with an atmosphere of N_2_ + CO_2_ (OD_600_: 0.343, 0.327, 0,431 and 0.31). Furthermore, the *Terrisporobacter* isolates HSE, ELB, DSL and COM produced considerably higher amounts of acetate after 96 h of incubation in cultures with a H_2_ + CO_2_ atmosphere (31.60 mM, 33.38 mM, 45.69 mM and 54.58 mM,) than in control cultures with an atmosphere of N_2_ + CO_2_ (17.44 mM, 19.07 mM, 23.42 mM and 15.64 mM). The *Terrisporobacter* isolate COM was the only isolate showing small amounts of ethanol (up to 2.92 mM) besides the main product acetate under a H_2_ + CO_2_ atmosphere. Ethanol production was not observed in N_2_ + CO_2_ control cultures and occurred in H_2_ + CO_2_ cultures mainly with the onset of the stationary growth phase, simultaneous to the production of acetate (Figure 4). The *Acetoanaerobium* isolate DSL2 showed considerably lower maximal growth values in H_2_ + CO_2_ cultures (OD_600_: 0.231) than in the N_2_ + CO_2_ control cultures (OD_600_: 0.379). DSL2 produced similar amounts of acetate after 96 h of incubation under a H_2_ + CO_2_ atmosphere (21.54 mM) than under the N_2_ + CO_2_ control atmosphere (24.36 mM) (Supplementary Figure S1). Growth of DSL2 in the N_2_ + CO_2_ control cultures stagnated after the initial 17 h of incubation, while H_2_ + CO_2_ cultures of DSL2 continued to grow until 48 h of incubation. The GC-measurements of all isolates under a H_2_ + CO_2_ and a N_2_ + CO_2_ atmosphere showed besides the main products acetate and ethanol trace amounts of other products. The concentration of these trace amounts did not considerably differ between cultures of both atmospheres and did not change over the course of incubation. These products were not further investigated.

#### 3.3.3 Prophage activity screening

**Figure 5:**
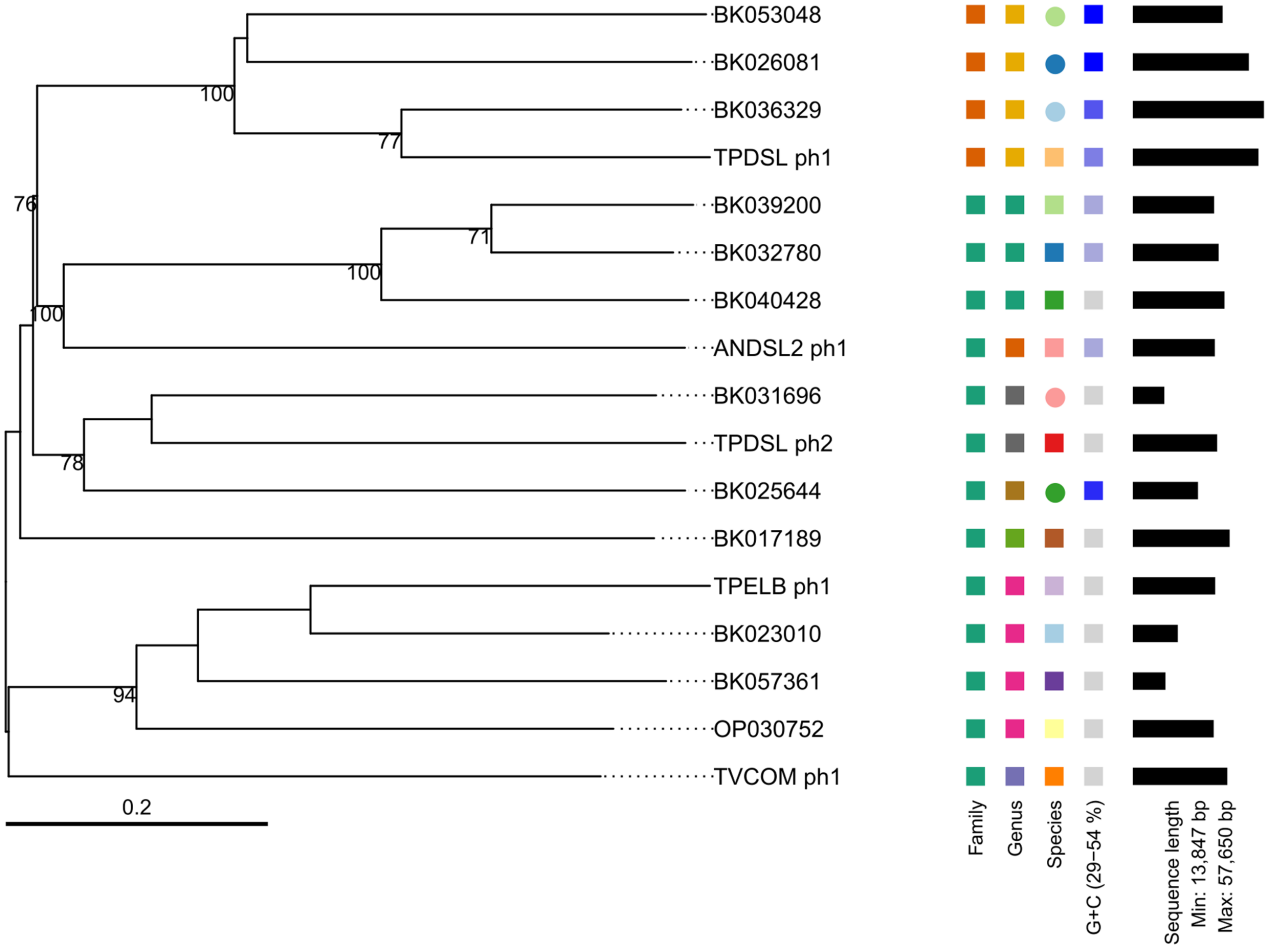
Phylogram of novel *Terrisporobacter* and *Acetoanaerobium* phage genomes and related phages. Branch lengths are scaled in terms of GBDP distance formula *d*_0_ and the black numbers below branches are confidence scores yielding an average branch support of 71%. The tree was rooted at the midpoint.

We performed a prophage activity screening for all of our novel isolates, in order to identify potential growth inhibitions by prophage activity. Isolate DSL showed two active prophage regions (Supplementary Figure S2). The first active prophage region (TPDSL_ph1) was 55 Kb in size and matched to the prediction of a complete prophage by PHASTEST and a genomic island predicted by IslandViewer4, while the second active prophage region (TPDSL_ph2) was 37 Kb in size and matched to another complete prophage region predicted by PHASTEST. Isolate ELB showed one active prophage region (TPELB_ph1) with a size of 36 Kb matching one of the two predicted prophage regions by PHASTEST (Supplementary Figure S3). A proportion of this active region was also predicted as a genomic island by IslandViewer4. Isolate COM showed one active prophage region (TVCOM_ph1) with a size of 42 Kb. The position aligned with two adjacent regions predicted as incomplete and complete prophages by PHASTEST (Supplementary Figure S4). Isolate HSE did not yield phage DNA in our activity screening. Isolate DSL2 showed an active prophage region with a size of 36 Kb (ANDSL2_ph1) matching the only predicted region of a complete prophage by PHASTEST (Supplementary Figure S5). All prophage genomes were annotated to encode genes assigned to the function classes of transcription regulation, lysis, integration and excision, head and packaging, connector and tail. Furthermore, every prophage region encoded genes of the class DNA/RNA and nucleotide metabolism. However, TPDSL_ph1 was the only prophage region encoding a DNA invertase flanked on both sides by an integrase. The TPDSL_ph1 prophage region was also the only region encoding a γ-glutamyl cyclotransferase assigned to the class moron, auxiliary metabolic gene and host takeover. The γ-glutamyl cyclotransferase was preceded by two genes annotated as amidoligase, which were also only identified in the TPDSL_ph1 prophage. We did not detect significant hits to reference genomes of phage isolates in the viral databases of NCBI Virus or PhageScope for our novel phage genomes. BLASTp search of the sequence of the large terminase subunit of the novel phage genomes identified several hits to metagenomic assembled phage genomes (Table 5). PhageScope predicted the novel phage genomes and metagenomic assembled phage genomes to be affiliated to the class of *Caudoviricetes*. Interestingly, *Clostridioides difficile* was the only host predicted to also belong to the *Peptostreptococcaceae* family, like *Terrisporobacter* or *Acetoanaerobium*. The phages TPELB_ph1, TVCOM_ph1, TPDSL_ph2 and ANDSL2_ph1 were all predicted to employ a temperate lifestyle, only TPDSL_ph1 was predicted to be a virulent bacteriophage. The whole genome-based phylogram created by VICTOR placed the potential relatives into the same phylogenomic group in all cases, with the only exception of the TVCOM_ph1 genome not clustering with the BK017189 phage genome (Figure 5). The phylogenomic groups of TPELB_ph1, TVCOM_ph1, TPDSLph2 and ANDSL2_ph1 were assigned to the same phage family of different phage genera, while the phylogenomic group of TPDSL_ph1 was assigned to another phage family.

**Table 5:** Related prophage genomes of the novel phage genomes derived from the prophage activity screening identified by BLASTp search of the protein sequence of the large terminase subunit.

Phage	Query	Query large terminase coverage (%)	Query large terminase identity (%)	GC (%)	Length (bp)	Genes	Completeness score (quality)	Host prediction	Lifestyle prediction	Taxonomy prediction
TPELB_ph1	TPELB_ph1	–	–	28.82	36,206	58	High	*Intestinibacter bartlettii*	temperate	*Caudoviricetes*
	BK057361	98	80.37	29.27	14,335	16	Low	*Clostridium saudiense*	virulent	*Caudoviricetes*
	BK023010	98	80.10	29.23	19,760	28	Medium	*Clostridium saudiense*	temperate	*Caudoviricetes*
	OP030752	100	79.97	30.97	35,545	49	Medium	*Intestinibacter bartlettii*	temperate	*Caudoviricetes*
TVCOM_ph1	TVCOM_ph1	–	–	28.90	41,547	55	High	*Paraclostridium bifermentans*	temperate	*Caudoviricetes*
	BK017189	98	77.45	28.95	42,555	75	High	*Clostridium perfringens*	temperate	*Caudoviricetes*
TPDSL_ph1	TPDSL_ph1	–	–	37.11	55,229	60	High	*Anaerostipes hadrus*	virulent	*Caudoviricetes*
	BK053048	100	92.31	52.5	39,484	45	Medium	*Clostridioides difficile*	temperate	*Caudoviricetes*
	BK026081	99	92.28	54.24	51,033	56	High	*Butyricicoccus pullicaecorum*	temperate	*Caudoviricetes*
	BK036329	100	92.31	40.33	57,650	63	High	*Anaerostipes hadrus*	virulent	*Caudoviricetes*
TPDSL_ph2	TPDSL_ph2	–	–	29.16	37,068	54	Medium	*Intestinibacter bartlettii*	temperate	*Caudoviricetes*
	BK031696	100	86.96	31.08	13,847	19	Low	*Clostridioides difficile*	temperate	*Caudoviricetes*
	BK025644	100	71.25	43.09	28,645	37	Medium	*Blautia producta*	virulent	*Caudoviricetes*
ANDSL2_ph1	ANDSL2_ph1	–	–	31.73	36,061	61	High	*Roseburia inulinivorans*	temperate	*Caudoviricetes*
	BK040428	98	46.40	30.69	40,302	61	High	*Anaerostipes hadrus*	temperate	*Caudoviricetes*
	BK039200	97	48.11	32.43	35,752	56	Medium	*Salmonella enterica*	temperate	*Caudoviricetes*
	BK032780	97	48.48	32.00	37,733	64	Medium	*Salmonella enterica*	temperate	*Caudoviricetes*

The table shows the large terminase coverage and identity in percentage of BLASTp hits to the respective large terminase of the novel phage genomes and the corresponding PhageScope host, lifestyle and taxonomy predictions.

## 4 Discussion

### 4.1 Genomic characterization

The *Terrisporobacter* isolates HSE, ELB, DSL, and COM showed genome sizes of 4 to 4.1 Mb and a GC-content of 29%, which is in line with the available genomes of other *Terrisporobacter* members. Plasmids were reported for the circular high-quality genomes of *T. hibernicus* NCTC14625^T^, *T, glycolicus* DSM 1288^T^, *T. mayombei* DSM 6539^T^ and *T. petrolearius* JCM 19845^T^ (Böer et al., 2023; Mitchell et al., 2023). The plasmids pTPHSE2 (21.696 Kb), pTPELB1 (21.684 Kb), pTPDSL1 (21.645 Kb) and pTVTCOM1 (21.519 Kb) showed the genetic structure of the *Terrisporobacter*-specific plasmids encoding genes for the proline-dependent reductive branch of the Stickland reaction (Böer et al., 2023). The pTVTCOM2 plasmid of *T. vanillatitrophus* encoded *uviA/B* genes, which were shown to be involved in the UV-inducible production of bacteriocin Bcn5 in *Clostridium perfringens* or the production of toxins in *Clostridioides difficile*, *Clostridium tetani* or *Clostridium botulinum* (Dupuy et al., 2005). Other plasmids detected in the *Terrisporobacter* isolates were unique for the respective isolate and only hypothetical proteins were encoded. ANIm analysis showed a phylogenomic cluster applying a species threshold of 95% (Goris et al., 2007) of the *Terrisporobacter* strains HSE, JCM 19845^T^, NCTC 14625^T^, DSL, KPPR-9, WW3900, UN03-225, FS03 and ELB with ANIm values ranging from 96.0 to 99.0%. The dDDH (d_4_) comparisons between those *Terrisporobacter* strains confirmed the phylogenomic cluster with values ranging from 84.0 to 91.2%, with the exception of the strains HSE and JCM 19845^T^ where multiple comparisons fell below the dDDH species threshold of 70%. In such cases it is recommended to consider the confidence intervals of the *in silico* dDDH (d_4_) predictions (Meier-Kolthoff et al., 2013). These ranged across the species threshold of 70% with confidence scores ranging from 63.6 to 74.1% for the dDDH (d_4_) values of strains HSE and JCM 19845^T^ (Supplementary Table S3). Furthermore, as we are comparing circular high-quality genomes, we can also consult the dDDH values calculated by the d_0_ and d_6_ formula considering the genome length, and not only the d_4_ formula, which performs most robust for draft genomes as it is independent on genome lengths (Meier-Kolthoff et al., 2013). The dDDH values of strains HSE and JCM 19845^T^ compared to the other strains of the phylogenomic cluster ranged from 70.4 to 85.1% for formula d_0_ and 72.0 to 85.1% for formula d_6_ supporting the phylogenomic clustering of the ANIm analysis (Supplementary Table S3). Therefore, we propose to reclassify and summarize the *Terrisporobacter* isolates HSE, JCM 19845^T^, NCTC 14625^T^, DSL, KPPR-9, WW3900, UN03-225, FS03 and ELB under one species name. As the species *T. petrolearius* was described prior to *T. hibernicus*, we propose to classify all genomes and the corresponding strains of this phylogenomic cluster as *T. petrolearius*. The closest relative of the COM isolate was *T. mayombei* DSM 6539^T^ but the ANIm and dDDH values remained under the corresponding species thresholds in all comparisons. Hence, the COM isolate represented a novel *Terrisporobacter* species, which was further supported by the 599 unique OGs detected in this isolate and the whole genome-based phylogram created by TYGS. The name *Terrisporobacter vanillatitrophus* is proposed for the COM isolate. Several unique OGs of the COM isolate allowed to infer unique physiological characteristics of the novel *Terrisporobacter* species. For example, the unique OGs of ArgBCDFGHJ indicate a functional ornithine-based synthesis of arginine in *T. vanillatitrophus*, which was identified as incomplete in the genome-based metabolic analysis of the type strains of *T. glycolicus*, *T. petrolearius* and *T. mayombei* (Böer et al., 2023). The PFOR links catabolism and anabolism during autotrophic growth in acetogens and is encoded by one fused gene in every other *Terrisporobacter* genome (Furdui and Ragsdale, 2000; Katsyv et al., 2021; Böer et al., 2023). In contrast, besides the fused PFOR (TVTCOM_10920), the COM isolate encodes an additional PFOR encoded by four separate genes, which was hitherto reported to be characteristically present in methanogenic archaea (Blamey and Adams, 1993; Ma et al., 1997) or hyperthermophilic bacteria (Blamey and Adams, 1994; Eram et al., 2015). Besides the additional PFOR identified for the COM isolate, every *Terrisporobacter* isolate fulfilled all prerequisites for H_2_/CO_2_-dependent acetogenesis in line with the metabolic model described for the *Terrisporobacter* genus based on the type strains of *T. mayombei*, *T. petrolearius* and *T. glycolicus* (Böer et al., 2023). The model comprises a complete WLP-gene cluster, a potential HDCR-complex, an electron-bifurcating hydrogenase (HydABC), an electron-bifurcating *Sporomusa*-type Nfn transhydrogenase (StnABC) and a Rnf-complex (RnfABCDEG).

The *Acetoanaerobium* isolate DSL2, with a genome size of 2.6 Mb, was smaller than the type strains of *A. noterae* (2.8 Mb), *A. sticklandii* (2.7 Mb) and *A. pronyense* (3 Mb). The GC-content of 34% was slightly higher than that of other *Acetoanaerobium* genomes (33%). The closest relative of DSL2 was identified by ANIm and dDDH analyses as the type strain of *A. noterae*. Both strains, together with the type strain of *A. sticklandii*, formed a phylogenomic cluster with ANIm values ranging from 96.5 to 97.7% and dDDH (d_4_) values ranging from 70 to 78.6%. As these values fall into the respective species threshold of 95 and 70%, respectively, this phylogenomic cluster was identified to correspond to the same bacterial species. As *A. sticklandii* was described before *A. noterae*, we propose to reclassify all strains of this phylogenomic cluster to the species *A. sticklandii*. The only available *Acetoanaerobium* genome analyzed falling under the species thresholds of ANIm and dDDH was *A. pronyense* DSM 27512^T^, forming its own phylogenomic cluster. The structure of the WLP-gene cluster in all strains of the phylogenomic cluster of *A. sticklandii* resembled the structure described for different *Clostridium* or *Terrisporobacter* species (Poehlein et al., 2015; Böer et al., 2023), with the only difference that the gene encoding the bifunctional cyclohydrolase/dehydrogenase (FolD) was not encoded as part of the WLP-cluster, but located in another genomic region.

### 4.2 Physiological characterization

Members of the genus *Terrisporobacter* were described as anaerobic and Gram-positive bacteria, forming rod-shaped and motile cells producing terminal endospores (Gerritsen et al., 2014). These characteristics were met by all four obtained isolates assigned to the genus *Terrisporobacter*. Growth dependence on the addition of yeast extract or peptone has been described for the type strains of *T. mayombei* and *T. glycolicus* (Kane et al., 1991; Chamkha et al., 2001). *A. noterae* was described to form rod-shaped Gram-negative cells without endospore formation (Sleat et al., 1985), which was in line with the observed morphology of the DSL2 isolate and the isolation from a non-pasteurized enrichment culture. Although *A. noterae* was reported to stain Gram-negative, cells were described to form a two-layered cell wall structure atypical for Gram-negative bacteria (Sleat et al., 1985). *A. sticklandii* and *A. pronyense* were both described as Gram-positive bacteria (Stadtman and McClung, 1957; Bes et al., 2015).

The major cellular fatty acids detected in *T. vanillatitrophus* COM showed considerable differences to the composition of other *Terrisporobacter* type strains. With 19% of C_16:0_, the COM isolate contained lower proportions than *T. mayombei* (26.3%), *T. petrolearius* (23.6%) and *T. hibernicus* (22.8%). *T. glycolicus* contained similar proportions of C_16:0_ with 18.5%. The other major cellular fatty acids C16:1 ∆9 (7.3%) and C16:1 ∆7 (6%) detected for the COM isolate were identified in similar abundances for the other four *Terrisporobacter* type strains. DMA fatty acids were not reported by Deng et al. (2015) and Mitchell et al. (2023) for the type strains of *T. glycolicus*, *T. mayombei*, *T. petrolearius* and *T. hibernicus* (Deng et al., 2015). The proposal of the genus *Terrisporobacter* in 2014 by Gerritsen et al. reported lower values for C_16:0_ DMA for the type strains of *T. glycolicus* (9.3%) and *T. mayombei* (8.3%) than we identified in the COM isolate (Gerritsen et al., 2014). In summary, the major cellular fatty acid analysis of the COM isolate supported the classification as a novel *Terrisporobacter* species.

The physiological optima and substrates utilized by the novel *Terrisporobacter* isolates were generally consistent with the descriptions of the four type strains of the *Terrisporobacter* genus (Table 6). However, some unique physiological features of the novel isolates were identified. The isolates HSE and ELB showed lower temperatures and higher pH values for optimal growth than other *Terrisporobacter* isolates or reference strains, although the physiological optima may not be directly comparable due to methodological differences. ELB was the first *Terrisporobacter* strain identified to utilize trehalose for growth. The COM isolate utilized vanillate, mannitol and 1,2-propanediol for growth, which was not described for any of the type strains of the four *Terrisporobacter* species. The fermentation of mannitol and 1,2-propanediol by the COM isolate matched the respective unique OGs detected in the pan/core genome analysis. The degradation of glucose to acetate and ethanol by the COM isolate was in line with the detected products of sugar or ethylene glycol fermentation reported for the *T. glycolicus* DSM 1288^T^ type strain (Gaston and Stadtman, 1963) or the acetogenic *T. glycolicus* RD-1 strain (Küsel et al., 2001). The determined optimal growth conditions of the *A. noterae* DSL2 isolate were similar to the data reported for the type strains of *A. noterae* (Sleat et al., 1985) and *A. sticklandii* (Bes et al., 2015) (Table 7). Furthermore, DSL2 utilized glucose and maltose, which matched with the description of *A. noterae* ATCC 35199^T^. The *Acetoanaerobium* isolate DSL2 additionally fermented fructose, sorbitol, sucrose, trehalose, melibiose, raffinose, melezitose and dextrin. Growth with fructose, sorbitol, sucrose, trehalose and melezitose deviates from the description of the *A. noterae* type strain, utilization of the other substrates was not assessed for other type strains. Surprisingly, the *Acetoanaerobium* isolate DSL2 did not grow with DMG, although it was isolated from an enrichment culture containing DMG as substrate. As no other energy source was supplemented than yeast extract, the isolate was likely growing by pairwise fermentation of amino acids via the Stickland reaction in the enrichment culture (Stickland, 1935). Substrates identified to be used for growth by the isolates of this study were only identified to support growth when yeast extract and peptone were also supplemented. This was required due to the dependency on the addition of complex substrates for bacteria of the genera *Terrisporobacter* and *Acetoanaerobium*. However, as complex substrates were also supplemented during the characterization of *T. mayombei* (Kane et al., 1991), *T. glycolicus* (Chamkha et al., 2001), *T. petrolearius* (Deng et al., 2015), *A. noterae* (Sleat et al., 1985), and *A. pronyense* (Bes et al., 2015) the substrate utilization data of this study is comparable to the reported data of the respective reference strains.

**Table 6:** Results of the physiological characterization of the *T. petrolearius* isolates HSE, ELB, DSL, and the *T. vanillatitrophus* isolate COM in comparison to the type strain descriptions of the *Terrisporobacter* species *T. glycolicus* DSM 1288^T^, *T. mayombei* DSM 6539^T^, *T. petrolearius* JCM 19845^T^ and *T. hibernicus* NCTC 14625^T^.

Substrate	HSE	ELB	DSL	COM	DSM 1288^T*^	DSM 6539^T**^	JCM 19845^T***^	NCTC 14625^T****^
Gram-staining	positive	positive	positive	positive	positive	positive	positive	positive
Temperature optimum	25°C	25°C	30°C	35°C	37°C	33°C	40°C	35–40°C
Temperature range	20–45°C	20–45°C	20–40°C	20–40°C	20- < 45°C	15–45°C	15–45°C	20–40°C
pH optimum	8.5	8	7	7.5	7.4–7.6	n.r.	7–7.5	n.r.
NaCl optimum	0%	0%	0.1%	0.1%	n.r.	n.r.	n.r.	n.r.
NaCl range	0–3%	0–2%	0–5%	0–3%	0–<6.5%	n.r.	0–3%	0–5.5%
Xylose	−	−	+	+	+	+	+	?
Fructose	+	+	+	+	+	+	+	n.r.
Glucose	+	+	+	+	+	+	+	+
Glycerol	−	−	+	−	−	+	−	+
Sorbitol	+	+	+	+	+	+	+	+
Mannitol	−	−	−	+	−	−	−	−
Sucrose	+	+	−	−	−	−	−	−
Cellobiose	−	−	−	+	−	+	+	−
Trehalose	−	+	−	−	−	−	−	−
Maltose	+	+	+	+	+	+	+	+
Melibiose	−	−	−	−	−	−	+	n.r.
Raffinose	+	+	+	+	−	−	−	−
Melezitose	+	+	+	+	−	−	+	−
Dextrin	+	+	+	+	−	+	n.r.	n.r.
Starch	−	−	−	−	−	+	−	n.r.
Formate	−	−	−	+	n.r.	+	n.r.	n.r.
Pyruvate	−	−	−	−	+	+	n.r.	n.r.
Malate	−	−	−	−	n.r.	+	n.r.	n.r.
Vanillate	−	−	−	+	n.r.	n.r.	n.r.	n.r.
Syringate	−	−	−	−	n.r.	+	n.r.	n.r.
1,2-propanediol	−	−	−	+	n.r.	n.r.	n.r.	n.r.
H_2_/CO_2_	+	+	+	+	−	+	−	−

+, positive; −, negative; n.r., not reported;?, inconclusive. * data from Gaston and Stadtman (1963) and Chamkha et al. (2001). ** data from Kane et al. (1991). *** data from Deng et al. (2015). **** data from Mitchell et al. (2023).

**Table 7:** Results of the physiological characterization of the *A. sticklandii* isolate DSL2 in comparison to the type strain descriptions of the *Acetoanaerobium* species *A. noterae* ATCC 35199^T^, *A. sticklandii* DSM 519^T^, *A. pronyense* DSM 27512^T^.

Substrate	DSL2	ATCC 35199^T*^	DSM 519^T**^	DSM 27512^T***^
Gram-stain	Negative	Negative	Positive	Positive
Temperature optimum	35°C	37°C	30–37°C	35°C
Temperature range	35–50°C	n.r.	25–45°C	15–40
pH optimum	7	7.6	6.9	8.7
NaCl optimum (%)	0.1%	n.r.	0.5%	0.5%
NaCl range	0–7%	n.r.	0–7%	0–7%
D(−)-Ribose	−	−	+	+
D(−)-Fructose	+	−	−	−
D(+)-Galactose	−	−	−/+	+
D(+)-Glucose	+	+	+	+
D(−)-Sorbitol	+	−	n.r.	n.r.
D(+)-Sucrose	+	−	−/+	+
D(+)-Trehalose	+	−	−	+
D(+)-Maltose	+	+	+	+
D(+)-Melibiose	+	n.r.	n.r.	n.r.
D(+)-Raffinose	+	n.r.	n.r.	n.r.
D(+)-Melezitose	+	−	n.r.	n.r.
Dextrin	+	n.r.	n.r.	n.r.
Pyruvate	−	−	+	+
H_2_/CO_2_	−/+	+	−	−

+, positive; −, negative; −/+, inconclusive results; n.r., not reported. * data from Sleat et al. (1985). ** data from Stadtman and McClung (1957) and Bes et al. (2015). *** data from Bes et al. (2015).

All *Terrisporobacter* isolates shared a common growth pattern in H_2_ + CO_2_-supplemented cultures. Initial growth took place by consuming nutrients provided by the complex substrates peptone and yeast extract in the medium, likely by performing the acetate-producing pairwise fermentation of amino acids via the Stickland reaction (Stickland, 1935; Sangavai and Chellapandi, 2019). The trace amounts of other products besides acetate and ethanol detected by the GC-measurements likely originated from these Stickland reactions. *T. mayombei* was reported to produce trace amounts of isovalerate from yeast extract (Kane et al., 1991) and *T. glycolicus* was reported to produce trace amounts of isovalerate from yeast extract, casamino acids, trypticase and peptone (Chamkha et al., 2001). We hypothesize that the H_2_-producing Stickland reaction is inhibited by high partial pressures of H_2_, leading to lower initial growth of some isolates supplied with H_2_ + CO_2_ than in control cultures. The initial heterotrophic growth was then followed by the stationary growth phase, in which continuous acetate production was observed in H_2_-supplemented cultures of the *Terrisporobacter* isolates indicative of acetogenesis. This growth pattern on H_2_ + CO_2_ has been reported for the acetogenic type strain of *T. mayombei* and CO_2_-fixation via the WLP into acetate was verified by ^14^C isotopic labelling of CO_2_ (Kane et al., 1991). *T. vanillatitrophus* COM was the only *Terrisporobacter* isolate found to produce besides acetate also ethanol during the stationary growth phase in H_2_-supplemented cultures. From our experiments, we could not exclude the possibility that this ethanol was produced from the provided complex substrates via the Stickland reaction, however, the ethanol production coincided with the onset of the stationary growth phase where these substrates were already consumed. Furthermore, there was no ethanol production observed in the control cultures. There are two hypothetical routes for the production of ethanol by acetogenesis with H_2_ + CO_2_ in *T. vanillatitrophus*. The ethanol precursor acetaldehyde could be produced from reabsorbed acetate by an acetaldehyde ferredoxin oxidoreductase (Aor) or directly from acetyl-CoA using an acetaldehyde dehydrogenase (Aldh) (Daniell et al., 2012). Synthesis of ethanol could then occur via alcohol dehydrogenases (Adh). We detected a total of three possible *aor* genes (TVTCOM_11530, TVTCOM_02900 and TVTCOM_02970) and three bifunctional acetaldehyde/alcohol dehydrogenase genes (*adhE*; TVTCOM_05440, TVTCOM_30030 and TVTCOM_30090) encoded by the *T. vanillatitrophus* genome. Other putative alcohol dehydrogenases identified comprised genes encoding a long-chain-alcohol dehydrogenase 1 (*adh1*; TVTCOM_02640), an alcohol dehydrogenase 2 (*adhB*; TVTCOM_24670) and an aldehyde dehydrogenase (*aldH1*; TVTCOM_18480).

In summary, the physiological characterizations of the COM isolate showed considerable differences in comparison to the data of other *Terrisporobacter* type strains and thereby further supported the classification of the COM isolate from the genomic characterization as a novel *Terrisporobacter* species.

In contrast to the *Terrisporobacter* isolates, the *A. sticklandii* isolate DSL2 did not achieve higher maximal growth values in H_2_-supplemented cultures than in the control cultures. This was also reported for the acetogenic type strain of *A. noterae*. However, *A. noterae* was found to produce more than 4 times the amount of acetate in H_2_-supplemented cultures compared to control cultures (Sleat et al., 1985). Hence, we could not confirm acetogenesis of the DSL2 isolate in our experiments, as we observed equal amounts of acetate being formed in H_2_-supplemented cultures compared to control cultures. It has to be considered that the acetate measurements of *A. noterae* were also conducted after longer incubation times (376 h) than in our experiments (96 h). Some H_2_-supplemented cultures of the *A. noterae* type strain were reported to continue utilizing H_2_ for more than 100 days after entering the stationary growth phase (Sleat et al., 1985). Therefore, the DSL2 isolate requires follow-up experiments with prolonged incubation times to verify the continuous production of acetate in H_2_-supplemented cultures. These experiments should also include the *A. sticklandii* type strain.

Active prophage regions were identified, under the tested conditions in every novel isolate screened with the exception of the HSE isolate. The size of the inferred phage genomes (36–55 kb) and the identification of multiple tail-associated genes indicated an affiliation to the class of *Caudoviricetes* (Zrelovs et al., 2020), which was also supported by the PhageScope taxonomy prediction. The DNA invertase uniquely identified in TPDSL_ph1 may represent a tool acting as a genetic switch for the alternate expression of gene sets as described for the Gin DNA invertase of the *Escherichia coli* K12 phage Mu controlling host specificity (van de Putte et al., 1980). Furthermore, TPDSL_ph1 contained two amidoligases in proximity to a γ-glutamyl cyclotransferase, which were predicted to potentially modify cell walls by linking novel peptides in the enterobacteriophage phiEco32 and thereby preventing host accession by other phages (Iyer et al., 2009). To the best of our knowledge this is the first report of active prophages of bacteria from the genus *Terrisporobacter* and *Acetoanaerobium*. The absence of comparable phage genomes in viral databases underlines the novelty of these bacteriophages. Related phage genomes could be identified by comparisons of the sequence of the terminase large subunit and a whole genome-based phylogram indicated the presence of two different novel phage families and a total of eight different phage genera.

### 4.3 Description of *Terrisporobacter vanillatitrophus* sp. nov

*Terrisporobacter vanillatitrophus* (va.nil.la.ti.tro’phus. N.L. masc. n. vanillas, vanillate; Gr. masc. Adj. trophos, feeder; N.L. masc. Adj. vanillatitrophus, growing with vanillate). Cells are Gram-positive rods and produce terminal endospores. Growth occurs only under strict anaerobic conditions and is dependent on the addition of yeast extract. Growth ranges from the temperatures 20 to 40°C (optimum 35°C) and the NaCl concentrations 0 to 3% (optimum 0.1%). Optimal growth occurs at pH 7.5. The best growth under the tested conditions occurs with glucose as substrate, which is fermented to acetate and ethanol as fermentation products. Other substrates utilized for growth comprise xylose, fructose, sorbitol, mannitol, cellobiose, maltose, raffinose, melezitose, dextrin, formate, vanillate, 1,2-propanediol and H_2_ + CO_2_. Growth on H_2_ + CO_2_ occurs by initial heterotrophic growth followed by the stationary growth phase, where continuous acetate production is indicative of H_2_-dependent acetogenesis. H_2_-supplemented cultures also show the production of small amounts of ethanol in the stationary growth phase. Does not grow with arabinose, ribose, galactose, mannose, rhamnose, glucuronic acid, glycerol, inositol, sucrose, trehalose, lactose, melibiose, dextran, starch, pyruvate, lactate, malate, citrate, DMG, betaine, syringate, methanol, ethanolamine and CO under the tested conditions. The predominant cellular fatty acids are C16:0 (19%), C16:0 DMA (12.4%), C16:1 ∆9 (7.3%) and C16:1 ∆7 (6%). The type strain genome comprises a chromosome with the size of 4 Mb and three plasmids with the sizes of 21.5 Kb, 19.5 Kb and 5.6 Kb. The GC-content averages to 29%. The type strain COM was isolated from a compost sample taken from the Königsbühl composting plant near Göttingen, Germany. The Genbank/EMBL/DDBJ accession number of the 16S rRNA gene is PP196406. The type strain of *T. vanillatitrophus* is COM^T^ and was deposited under the identifiers DSM 116160^T^ and CCOS 2104^T^.

## 5 Conclusion

The novel acetogenic *Terrisporobacter* isolates expand the pool of available acetogens for biotechnological applications, in particular the novel species *T. vanillatitrophus* producing in addition to acetate also ethanol in H_2_-supplemented cultures. We propose to classify all isolates of the *T. petrolearius*/*T. hibernicus* phylogenomic cluster to the species *T. petrolearius* and all isolates of the *A. noterae/A. sticklandii* to the species *A. sticklandii*. Our results indicate that acetogenesis could be a more frequent trait of *Terrisporobacter* and *Acetoanaerobium* strains than hitherto described. However, these require follow-up experiments including all isolated strains of the respective genera. We could show that acetogenesis characteristically occurs in acetogenic strains from the genus *Terrisporobacter* by an initial heterotrophic growth phase on complex substrates followed up by a stationary growth phase with continuous acetate production. Furthermore, we provide first insights into active prophages in acetogenic bacteria from the genera of *Terrisporobacter* and *Acetoanaerobium*.

## Data availability statement

The datasets presented in this study can be found in online repositories. The names of the repository/repositories and accession number(s) can be found in the article/Supplementary material.

## Author contributions

TB: Conceptualization, Funding acquisition, Methodology, Writing – review & editing, Data curation, Formal analysis, Investigation, Visualization, Writing – original draft. MS: Investigation, Resources, Writing – review & editing. AL: Investigation, Writing – review & editing. LeE: Investigation, Writing – review & editing. JD: Investigation, Writing – review & editing. MH: Investigation, Writing – review & editing. LiE: Data curation, Investigation, Writing – review & editing. MB: Funding acquisition, Investigation, Writing – review & editing. RD: Conceptualization, Funding acquisition, Project administration, Supervision, Writing – review & editing. AP: Conceptualization, Funding acquisition, Methodology, Supervision, Writing – review & editing.
