# Supplementary material for: Isolation and characterization of novel acetogenic strains of the genera Terrisporobacter and Acetoanaerobium
Source: Front Microbiol. 2024 Jul 3;15:1426882. doi: 10.3389/fmicb.2024.1426882 (PMC11253131; doi:10.3389/fmicb.2024.1426882)
Supplement: Supplementary file 1 [file Data_Sheet_1.zip › Supplementary Material.pdf]

Supplementary Material

Isolation and characterization of novel acetogenic strains of the genera *Terrisporobacter* and *Acetoanaerobium*

Tim Böer [1], Miriam Antonia Schüler [1], Alina Lüschen [1], Lena Eysell [1], Jannina Dröge [1], Melanie Heinemann [1], Lisa Engelhardt [2], Mirko Basen [2], Rolf Daniel [1], Anja Poehlein [1*]

*** Correspondence:** Anja Poehlein
anja.poehlein@biologie.uni-goettingen.de

Supplementary Figures and Tables

Table S1: Modified DSM 311c medium

NH_4Cl	0.50 g
$MgCl_2$ x 6 H_2O	0.413 g
$CaCl_2$ x 2 H_2O	0.25 g
NaCl	2.25 g
$FeCl_2$ x 4 H_2O solution (0.071% w/v in 0.1 N HCl)	2.00 ml
Trace element solution	1.00 ml
Yeast extract	2.00 g
Peptone	2.00 g
Na-resazurin solution (0.1% w/v)	0.50 ml
K_2HPO_4	0.35 g
KH_2PO_4	0.23 g
Na_2CO_3	1.00 g
Wolins vitamin solution (DSM 141)	10.00 ml
L-Cysteine-HCl x H_2O	0.30 g
Na_2S x 9 H_2O	0.30 g
Distilled water	986.5 ml

Dissolve ingredients (except phosphates, carbonate, vitamins, cysteine and sulfide) and sparge medium with 80% N_2 and 20% CO_2 gas mixture for 30 – 45 min to make it anoxic. Dispense medium under same gas atmosphere into anoxic Hungate-type tubes or serum vials and autoclave. Add phosphates, vitamins (sterilized by filtration), cysteine and sulfide to the medium after autoclaving from sterile stock solutions prepared under 100% N_2 gas and carbonate from a sterile anoxic stock solution prepared under 80% N_2 and 20% CO_2 gas mixture. Adjust pH of complete medium to pH 7.0, if necessary.

Trace element solution

HCl (25%; 7.7 M)	10.00 ml
$FeCl_2$ x 4 H_2O	1.50 g
$ZnCl_2$	70.00 mg
$MnCl_2$ x 4 H_2O	100.00 mg
H_3BO_3	6.00 mg
$CoCl_2$ x 6 H_2O	190.00 mg
$CuCl_2$ x 2 H_2O	2.00 mg
$NiCl_2$ x 6 H_2O	24.00 mg
Na_2MoO_4 x 2 H_2O	36.00 mg
Na_2SeO_3 x 5 H_2O	3.00 mg
Na_2WO_4 x 2 H_2O	4.0 mg
Distilled water	990.00 ml

First dissolve $FeCl_2$ in the HCl, then dilute in water, add and dissolve the other salts. Finally make up to 1000.0 ml.

Wolins vitamin solution (DSM 141):

Biotin	2.00 mg
Folic acid	2.00 mg
Pyridoxine hydrochloride	10.00 mg
Thiamine HCl	5.00 mg
Riboflavin	5.00 mg
Nicotinic acid	5.00 mg
Calcium D-(+)-pantothenate	5.00 mg
Vitamin B_{12}	0.10 mg
p-Aminobenzoic acid	5.00 mg
(DL)-alpha-Lipoic acid	5.00 mg
Distilled water	1000.00 ml

Table S2: Accession numbers of analyzed *Terrisporobacter* and *Acetoanaerobium* reference genomes

Genome	Assembly accession (GenBank)
T. "muris" DSM 29186	GCA_024621905.1
T. "othiniensis" 08-306576	GCA_000808015.1
T. mayombei MSK.4.1	GCA_020740325.1
T. glycolicus DSM 1288^{T}	GCA_036812735.1
T. mayombei DSM 6539^{T*}	GCA_031202285.1
T. petrolearius JCM 19845^{T}	GCA_032598785.1
T. hibernicus NCTC 14625^{T}	GCA_020809405.1
T. glycolicus KPPR-9	GCA_900114105.1
T. glycolicus WW3900	GCA_021491335.2
T. petrolearius UN03-225	GCA_027675955.1
T. glycolicus FS03	GCA_017307015.1
A. noterae ATCC 35199^{T*}	GCA_900168025.1
A. sticklandii DSM 519^{T}	GCA_000196455.1
A. pronyense DSM 27512^{T}	GCA_017874355.1

* Isolates were H_2-dependent acetogenesis has been experimentally verified

Table S4: Cellular fatty acid analysis of *T. vanillatitrophus* COM and most closely related *Terrisporobacter* species. Fatty acid proportions are given in percentage. n.r., not reported; n.d., not detected.

Fatty acid	*T. vanillatitrophus* DSM 116160^{T}	*T. petrolearius* JCM 19845^{T*}	*T. glycolicus* DSM 1288^{T*}	*T. mayombei* DSM 6539^{T*}	*T. hibernicus* NCTC 14625^{T*}
$C_{11:0}$	0.1	n.r.	n.r.	n.r.	n.r.
Iso-$C_{11:0}$	0.4	n.r.	n.r.	n.r.	n.r.
Anteiso-$C_{11:0}$	0.2	n.r.	n.r.	n.r.	n.r.
$C_{12:0}$	0.7	2.6	2.4	2.4	2.1
Iso-$C_{12:0}$	0.3	n.r.	n.r.	n.r.	n.r.
$C_{13:0}$	0.3	n.r.	n.r.	n.r.	n.r.
Iso-$C_{13:0}$	0.4	n.r.	n.r.	n.r.	n.r.
Anteiso-$C_{13:0}$	0.1	n.r.	n.r.	n.r.	n.r.
$C_{14:0}$	3.6	4.5	3.0	4.1	3.9
$C_{14:0}$ DMA	0.7	n.r.	n.r.	n.r.	n.r.
Iso-$C_{14:0}$	1.4	2.6	2.3	1.9	2.1
$C_{14:1}$ $\Delta 5$	0.2	n.r.	n.r.	n.r.	n.r.
$C_{14:1}$ $\Delta 7$	0.4	n.r.	n.r.	n.r.	n.r.
$C_{14:1}$ $\Delta 7$ / $C_{15:0}$ ALDE	0.3	n.r.	n.r.	n.r.	n.r.
$C_{15:0}$	2.6	n.r.	n.r.	n.r.	n.r.
$C_{15:0}$ DMA	2.3-	n.r.	n.r.	n.r.	n.r.
$C_{15:1}$ $\Delta 6$	n.d.-	1.2	1.1	1.0	1.4
$C_{15:1}$ $\Delta 7$	0.4	n.r.	n.r.	n.r.	n.r.
$C_{15:1}$ $\Delta 8$	n.d.	2.3	1.9	1.4	2.3
$C_{15:1}$ $\Delta 9$	0.4	n.r.	n.r.	n.r.	n.r.
Iso-$C_{15:0}$	1.4	2.1	3.2	1.7	1.6

Iso-$C_{15:0}$ DMA	0.5	n.r.	n.r.	n.r.	n.r.
Iso-$C_{15:1}$ Δ8	0.2	n.r.	n.r.	n.r.	n.r.
Anteiso-$C_{15:0}$ DMA	0.2	n.r.	n.r.	n.r.	n.r.
$C_{16:0}$	19.0	23.6	18.5	26.3	22.8
$C_{16:0}$ DMA	12.4	n.r.	n.r.	n.r.	n.r.
$C_{16:0}$ ALDE	4.4	n.r.	n.r.	n.r.	n.r.
$C_{16:1}$ Δ5	n.d.	2.0	1.8	<1.0	1.0
$C_{16:1}$ Δ6+7	6.0 ($C_{16:1}$ Δ7)	9.6	8.3	7.8	6.5
$C_{16:1}$ Δ7 DMA	0.6	n.r.	n.r.	n.r.	n.r.
$C_{16:1}$ Δ9	7.3	8.1	7.4	6.5	6.4
$C_{16:1}$ Δ9 DMA	0.3	n.r.	n.r.	n.r.	n.r.
$C_{16:1}$ Δ11	0.8	n.r.	n.r.	n.r.	n.r.
Iso-$C_{16:0}$	3.4	5.1	6.4	4.0	3.6
Iso-$C_{16:0}$ DMA	2.1	n.r.	n.r.	n.r.	n.r.
Iso-$C_{16:0}$ H	n.d.	3.3	3.0	1.7	n.d.
Iso-$C_{16:0}$ ALDE	0.6	n.r.	n.r.	n.r.	n.r.
Iso-$C_{16:1}$ Δ10	1.1	n.r.	n.r.	n.r.	n.r.
$C_{17:0}$	4.8	5.9	6.0	6.1	9.2
$C_{17:0}$ DMA	1.5	n.r.	n.r.	n.r.	n.r.
$C_{17:1}$ Δ6	n.d.	5.2	5.5	3.0	4.8
$C_{17:1}$ Δ9	0.4	n.r.	n.r.	n.r.	n.r.
$C_{17:1}$ Δ11	2.8	n.r.	n.r.	n.r.	n.r.
Iso-$C_{17:0}$	0.9	1.2	2.8	1.5	1.4
Iso-$C_{17:0}$ DMA	0.5	n.r.	n.r.	n.r.	n.r.
Iso-$C_{17:0}$ Δ7+9	1.0	n.r.	n.r.	n.r.	n.r.
Iso-$C_{17:1}$ / Anteiso $C_{17:1}$	n.d.	n.d.	1.2	<1.0	5.9
Anteiso-$C_{17:0}$	0.7	<1.0	1.7	1.3	<1.0
Anteiso-$C_{17:0}$ DMA	0.6	n.r.	n.r.	n.r.	n.r.
$C_{18:0}$	1.3	3.2	1.4	6.0	1.58
$C_{18:0}$ DMA	0.2	n.r.	n.r.	n.r.	n.r.
$C_{18:1}$ Δ5	n.d.	4.3	5.2	3.6	5.1
$C_{18:1}$ Δ6+7	n.d.	3.6	3.7	6.0	2.5
$C_{18:1}$ Δ9	2.0	1.1	1.3	1.4	<1.0
$C_{18:1}$ Δ11	3.9	n.r.	n.r.	n.r.	n.r.
$C_{18:1}$ Δ11 DMA	0.1	n.r.	n.r.	n.r.	n.r.
$C_{18:1}$ Δ13	1.6	n.r.	n.r.	n.r.	n.r.
$C_{18:1}$ Δ? DMA	0.2	n.r.	n.r.	n.r.	n.r.
Iso-$C_{18:0}$	0.2	n.r.	n.r.	n.r.	n.r.
Iso-$C_{18:1}$ Δ11	0.3	n.r.	n.r.	n.r.	n.r.
Iso-$C_{18:1}$ H	n.d.	n.d.	2.2	n.d.	1.2
$C_{17:1}$ DMA/ Iso-$C_{18:1}$ Δ9	0.5	n.r.	n.r.	n.r.	n.r.

* data from [8]

DMA – dimethyl acetal
ALDE - aldehyde
Δ? – position of double-bond unclear

A

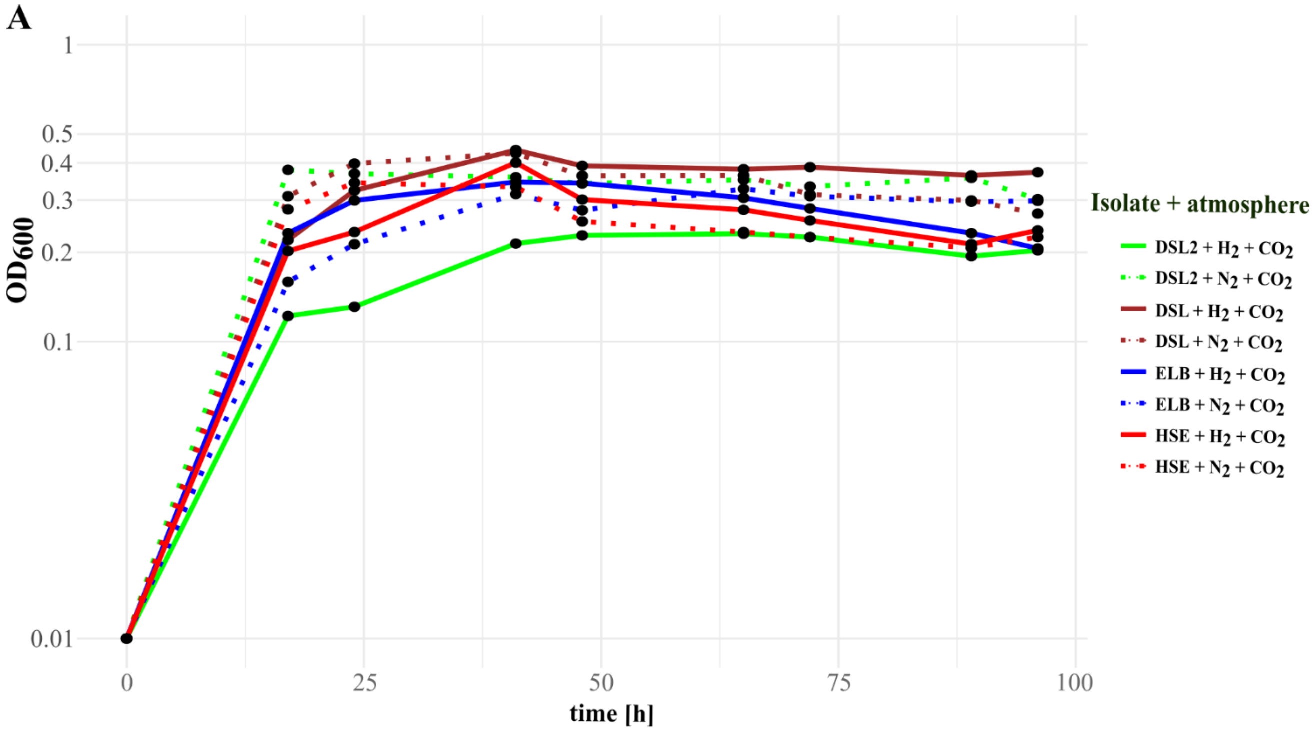

B

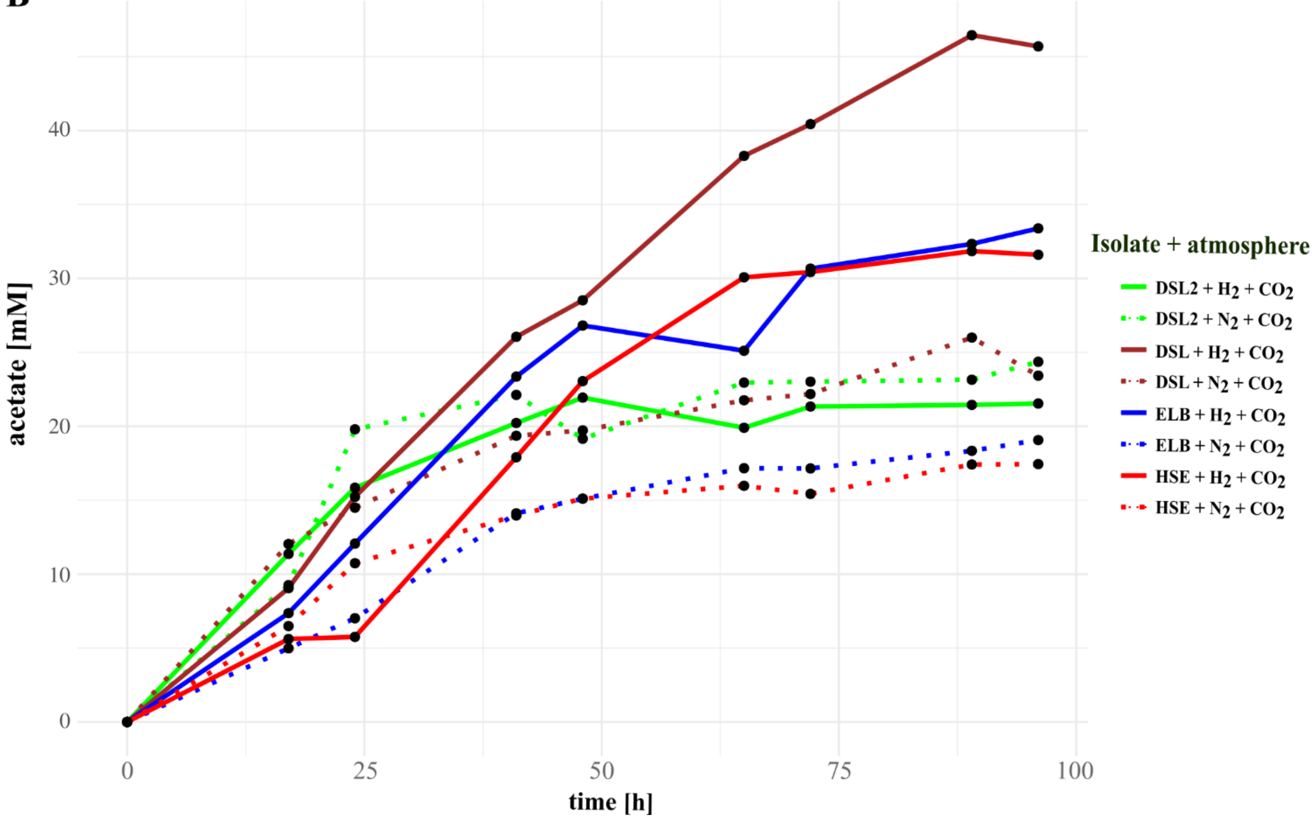

Figure S1. Growth in H_2-supplemented cultures and control cultures of the novel isolates ELB, HSE, DSL and DSL2. Measured OD_{600} growth values for isolates grown with an atmosphere of H_2 + CO_2 (solid lines) and control cultures with an atmosphere of N_2 + CO_2 (dotted lines) (**A**). GC-measurements detecting the amount of produced acetate in H_2 + CO_2 (solid lines) and N_2 + CO_2 control cultures (dotted lines) (**B**). The color code for the isolates analyzed is shown on the right.

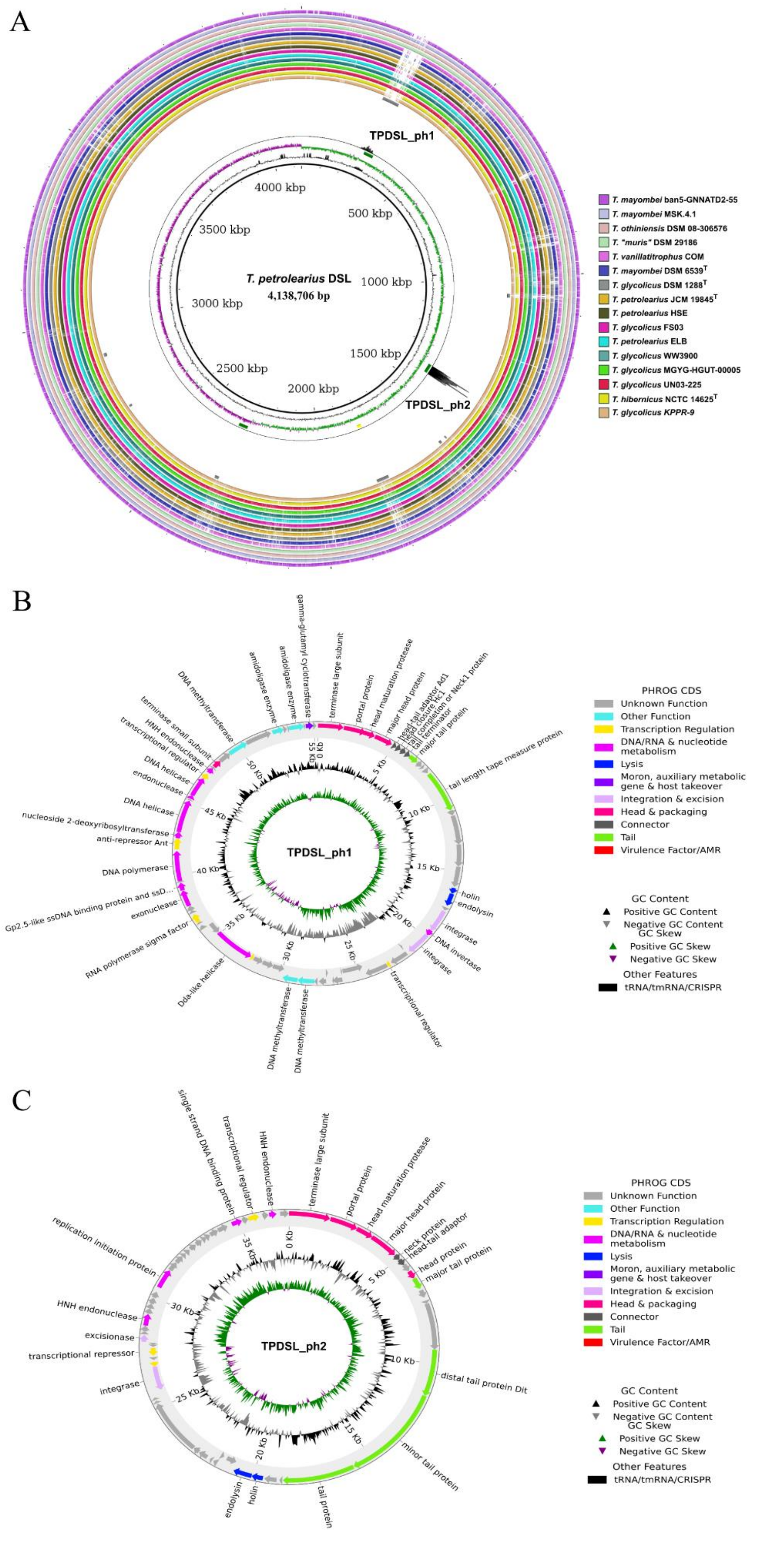
A
TPDSL_ph1
TPDSL_ph2
T. petrolearius DSL
4,138,706 bp
4000 kbp
500 kbp
3500 kbp
1000 kbp
3000 kbp
1500 kbp
2500 kbp
2000 kbp
T. mayombei ban5-GNNATD2-55
T. mayombei MSK.4.1
T. othiniensis DSM 08-306576
T. "muris" DSM 29186
T. vanillatitrophus COM
T. mayombei DSM 6539T
T. glycolicus DSM 1288T
T. petrolearius JCM 19845T
T. petrolearius HSE
T. glycolicus FS03
T. petrolearius ELB
T. glycolicus WW3900
T. glycolicus MGYG-HGUT-00005
T. glycolicus UN03-225
T. hibernicus NCTC 14625T
T. glycolicus KPPR-9
B
TPDSL_ph1
gamma-glutamyl cyclotransferase
amidoligase enzyme
amidoligase enzyme
terminase large subunit
portal protein
head maturation protease
major head protein
DNA methyltransferase
terminase small subunit
HNH endonuclease
transcriptional regulator
DNA helicase
endonuclease
DNA helicase
nucleoside 2-deoxyribosyltransferase
anti-repressor Ant
DNA polymerase
Gp2.5-like ssDNA binding protein and ssD...
exonuclease
RNA polymerase sigma factor
Dda-like helicase
DNA methyltransferase
DNA methyltransferase
transcriptional regulator
integrase
DNA invertase
integrase
holin
endolysin
tail length tape measure protein
major tail protein
PHROG CDS
Unknown Function
Other Function
Transcription Regulation
DNA/RNA & nucleotide metabolism
Lysis
Moron, auxiliary metabolic gene & host takeover
Integration & excision
Head & packaging
Connector
Tail
Virulence Factor/AMR
GC Content
Positive GC Content
Negative GC Content
GC Skew
Positive GC Skew
Negative GC Skew
Other Features
tRNA/tmRNA/CRISPR
C
TPDSL_ph2
single strand DNA binding protein
transcriptional regulator
HNH endonuclease
terminase large subunit
portal protein
head maturation protease
major head protein
neck protein
head-tail adaptor
head protein
major tail protein
distal tail protein Dit
minor tail protein
tail protein
holin
endolysin
integrase
transcriptional repressor
excisionase
HNH endonuclease
replication initiation protein
PHROG CDS
Unknown Function
Other Function
Transcription Regulation
DNA/RNA & nucleotide metabolism
Lysis
Moron, auxiliary metabolic gene & host takeover
Integration & excision
Head & packaging
Connector
Tail
Virulence Factor/AMR
GC Content
Positive GC Content
Negative GC Content
GC Skew
Positive GC Skew
Negative GC Skew
Other Features
tRNA/tmRNA/CRISPR

Figure S2. Prophage activity screening of the novel *Terrisporobacter* isolate DSL. Visualization of the host genome in comparison to available *Terrisporobacter* genomes with BLASTn (**A**). The order of the inner rings is: host reference genome (black), GC-content of host genome (black), positive (purple) and negative (green) GC-skew, prophage prediction by PHASTEST (red, incomplete; yellow, questionable; green, intact), coverage graph (0-4900) of sequenced DNA from isolated phage particles (black), genomic islands prediction by IslandViewer4 (grey). The legend for the color code of the rings showing the BLASTn comparison is shown on the right. Inferred TPDSL_ph1 and TPDSL_ph2 phage genome sequences (**B** and **C**). The color code for the annotated phage genomes are shown on the right.

A

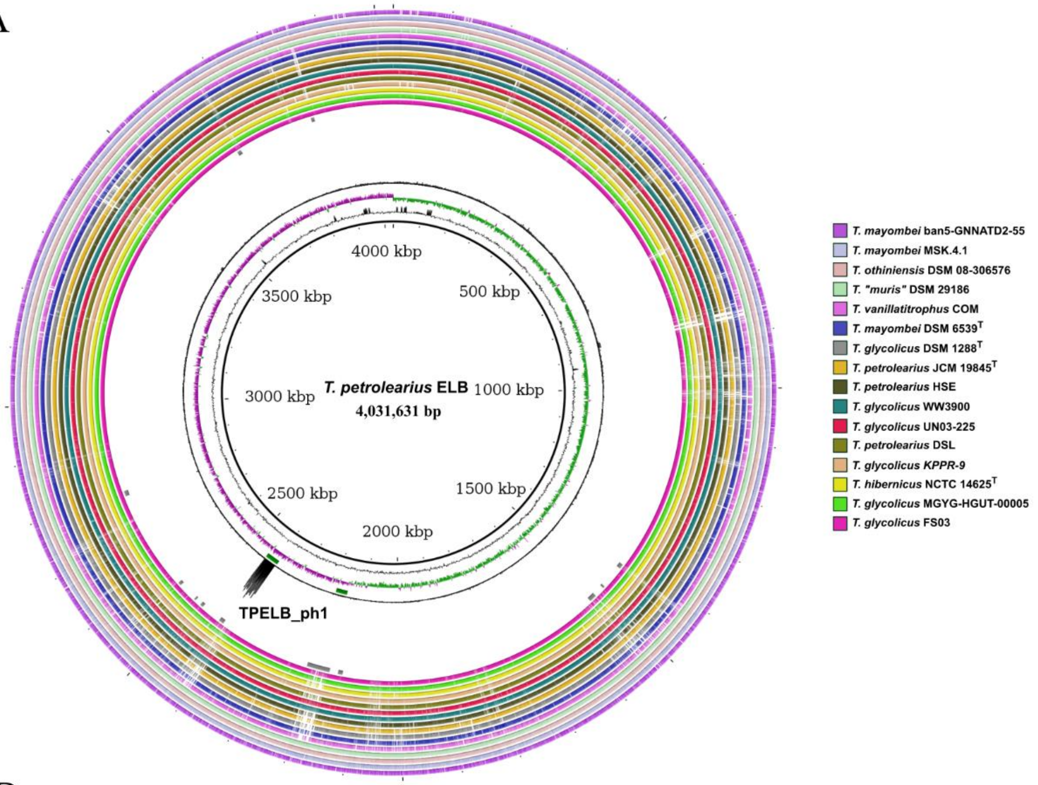

B

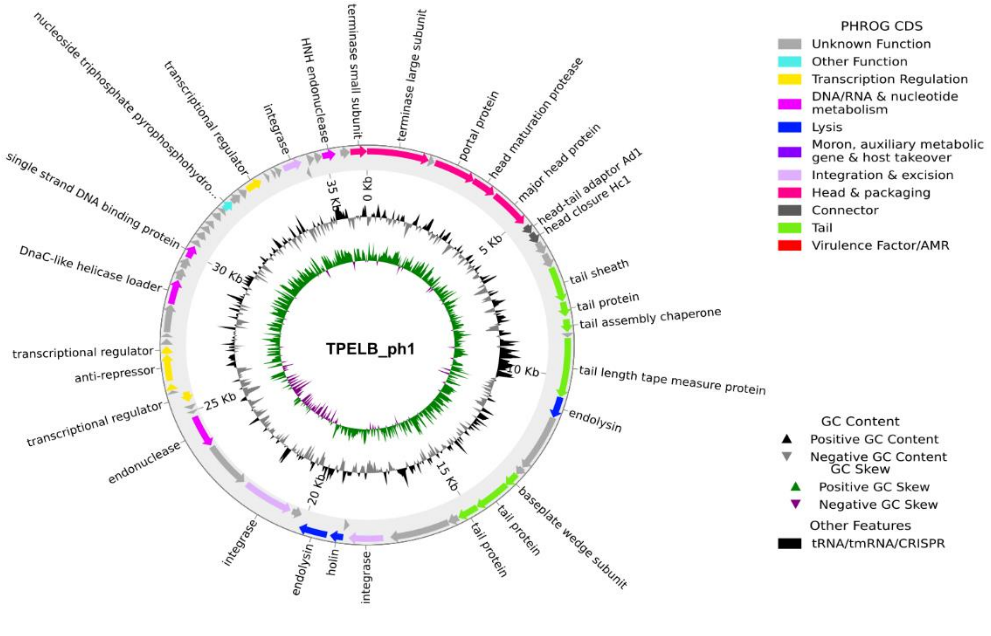

Figure S3. Prophage activity screening of the novel *Terrisporobacter* isolate ELB. Visualization of the host genome in comparison to available *Terrisporobacter* genomes with BLASTn (**A**). The order of the inner rings is: host reference genome (black), GC-content of host genome (black), positive (purple) and negative (green) GC-skew, prophage prediction by PHASTEST (red, incomplete; yellow, questionable; green, intact), coverage graph (0-1000) of sequenced DNA from isolated phage particles (black), genomic islands prediction by IslandViewer4 (grey). The legend for the color code of the rings showing the BLASTn comparison is shown on the right. Inferred TPELB_ph1 phage genome sequence (**B**). The color code for the annotated phage genome is shown on the right.

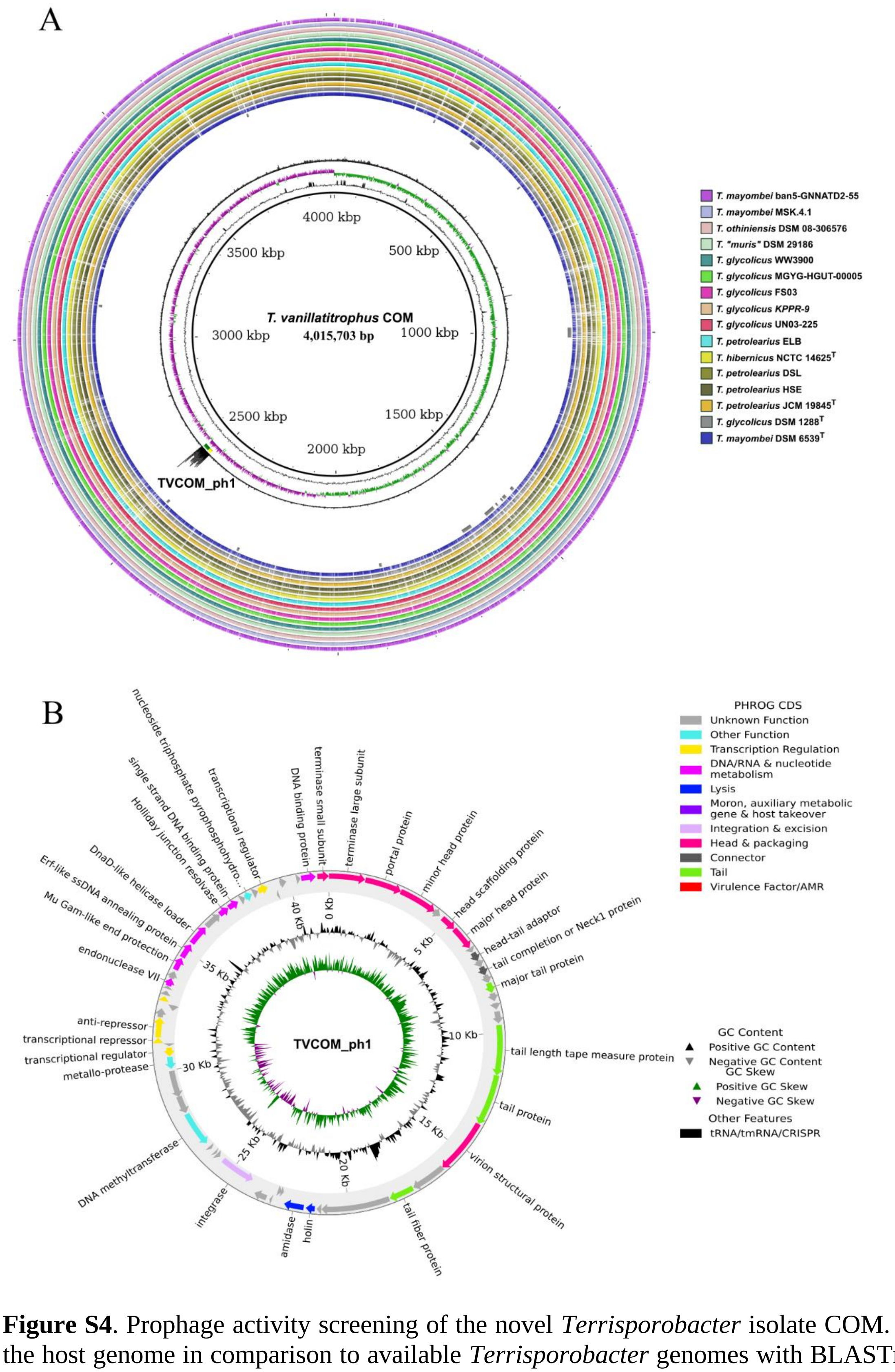

Figure S4. Prophage activity screening of the novel *Terrisporobacter* isolate COM. Visualization of the host genome in comparison to available *Terrisporobacter* genomes with BLASTn (**A**). The order of the inner rings is: host reference genome (black), GC-content of host genome (black), positive (purple) and negative (green) GC-skew, prophage prediction by PHASTEST (red, incomplete; yellow, questionable; green, intact), coverage graph (0-1000) of sequenced DNA from isolated phage particles

(black), genomic islands prediction by IslandViewer4 (grey). The legend for the color code of the rings showing the BLASTn comparison is shown on the right. Inferred TVCOM_ph1 phage genome sequence (**B**). The color code for the annotated phage genome is shown on the right.

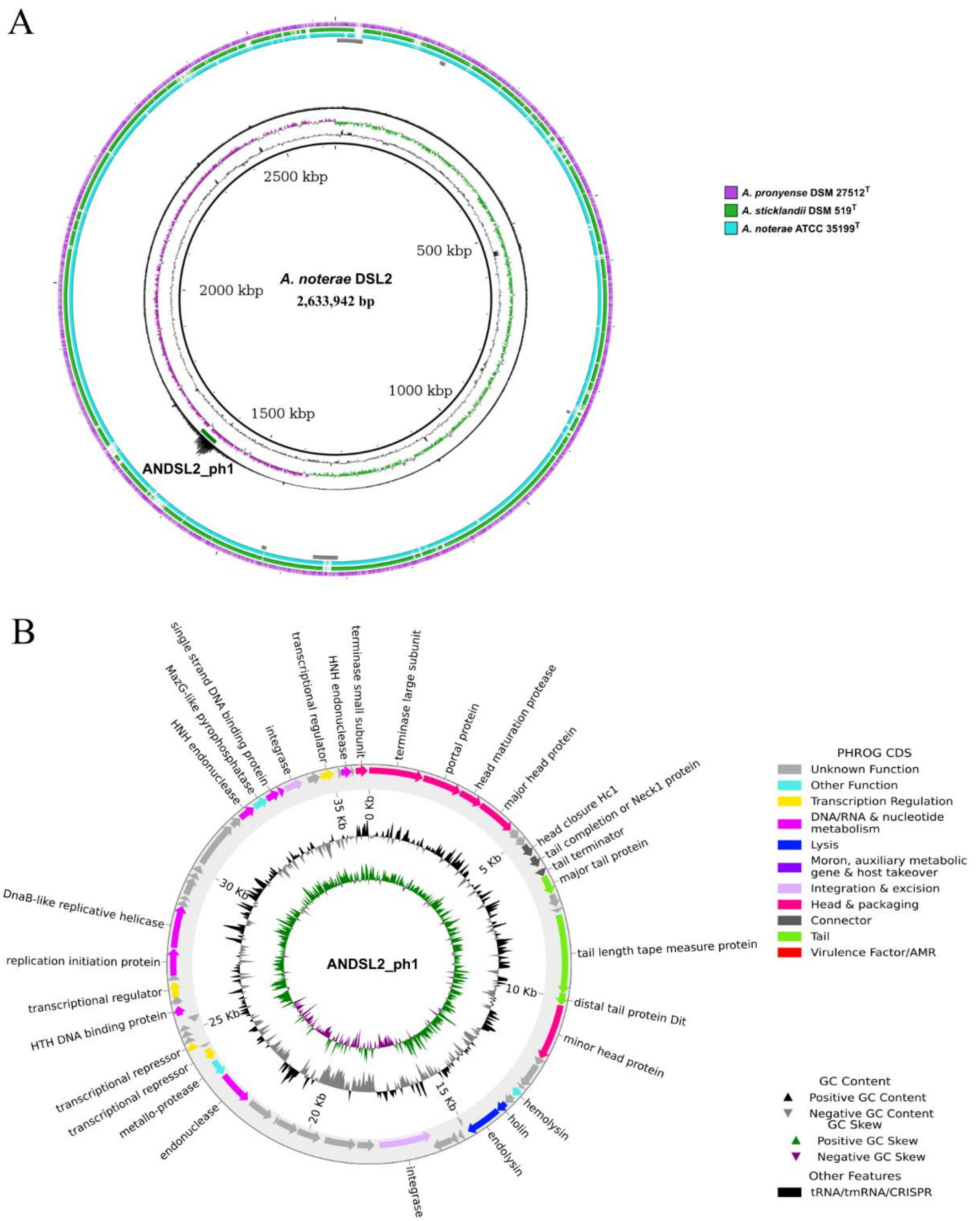

Figure S5. Prophage activity screening of the novel *Acetoanaerobium* isolate DSL2. Visualization of the host genome in comparison to available *Acetoanaerobium* genomes with BLASTn (**A**). The order of the inner rings is: host reference genome (black), GC-content of host genome (black), positive (purple) and negative (green) GC-skew, prophage prediction by PHASTEST (red, incomplete; yellow, questionable; green, intact), coverage graph (0-1000) of sequenced DNA from isolated phage particles (black), genomic islands prediction by IslandViewer4 (grey). The legend for the color code of the rings

showing the BLASTn comparison is shown on the right. Inferred ANDSL2_ph1 phage genome sequence (**B**). The color code for the annotated phage genome is shown on the right.
